# Muon reconstruction performance of the ATLAS detector in proton–proton collision data at $\sqrt{s}$=13 TeV

**DOI:** 10.1140/epjc/s10052-016-4120-y

**Published:** 2016-05-23

**Authors:** Atlas Collaboration, G. Aad, B. Abbott, J. Abdallah, O. Abdinov, B. Abeloos, R. Aben, M. Abolins, O. S. AbouZeid, N. L. Abraham, H. Abramowicz, H. Abreu, R. Abreu, Y. Abulaiti, B. S. Acharya, L. Adamczyk, D. L. Adams, J. Adelman, S. Adomeit, T. Adye, A. A. Affolder, T. Agatonovic-Jovin, J. Agricola, J. A. Aguilar-Saavedra, S. P. Ahlen, F. Ahmadov, G. Aielli, H. Akerstedt, T. P. A. Åkesson, A. V. Akimov, G. L. Alberghi, J. Albert, S. Albrand, M. J. Alconada Verzini, M. Aleksa, I. N. Aleksandrov, C. Alexa, G. Alexander, T. Alexopoulos, M. Alhroob, M. Aliev, G. Alimonti, J. Alison, S. P. Alkire, B. M. M. Allbrooke, B. W. Allen, P. P. Allport, A. Aloisio, A. Alonso, F. Alonso, C. Alpigiani, M. Alstaty, B. Alvarez Gonzalez, D. Álvarez Piqueras, M. G. Alviggi, B. T. Amadio, K. Amako, Y. Amaral Coutinho, C. Amelung, D. Amidei, S. P. Amor Dos Santos, A. Amorim, S. Amoroso, G. Amundsen, C. Anastopoulos, L. S. Ancu, N. Andari, T. Andeen, C. F. Anders, G. Anders, J. K. Anders, K. J. Anderson, A. Andreazza, V. Andrei, S. Angelidakis, I. Angelozzi, P. Anger, A. Angerami, F. Anghinolfi, A. V. Anisenkov, N. Anjos, A. Annovi, M. Antonelli, A. Antonov, J. Antos, F. Anulli, M. Aoki, L. Aperio Bella, G. Arabidze, Y. Arai, J. P. Araque, A. T. H. Arce, F. A. Arduh, J-F. Arguin, S. Argyropoulos, M. Arik, A. J. Armbruster, L. J. Armitage, O. Arnaez, H. Arnold, M. Arratia, O. Arslan, A. Artamonov, G. Artoni, S. Artz, S. Asai, N. Asbah, A. Ashkenazi, B. Åsman, L. Asquith, K. Assamagan, R. Astalos, M. Atkinson, N. B. Atlay, K. Augsten, G. Avolio, B. Axen, M. K. Ayoub, G. Azuelos, M. A. Baak, A. E. Baas, M. J. Baca, H. Bachacou, K. Bachas, M. Backes, M. Backhaus, P. Bagiacchi, P. Bagnaia, Y. Bai, J. T. Baines, O. K. Baker, E. M. Baldin, P. Balek, T. Balestri, F. Balli, W. K. Balunas, E. Banas, Sw. Banerjee, A. A. E. Bannoura, L. Barak, E. L. Barberio, D. Barberis, M. Barbero, T. Barillari, T. Barklow, N. Barlow, S. L. Barnes, B. M. Barnett, R. M. Barnett, Z. Barnovska, A. Baroncelli, G. Barone, A. J. Barr, L. Barranco Navarro, F. Barreiro, J. Barreiro Guimarães da Costa, R. Bartoldus, A. E. Barton, P. Bartos, A. Basalaev, A. Bassalat, R. L. Bates, S. J. Batista, J. R. Batley, M. Battaglia, M. Bauce, F. Bauer, H. S. Bawa, J. B. Beacham, M. D. Beattie, T. Beau, P. H. Beauchemin, P. Bechtle, H. P. Beck, K. Becker, M. Becker, M. Beckingham, C. Becot, A. J. Beddall, A. Beddall, V. A. Bednyakov, M. Bedognetti, C. P. Bee, L. J. Beemster, T. A. Beermann, M. Begel, J. K. Behr, C. Belanger-Champagne, A. S. Bell, G. Bella, L. Bellagamba, A. Bellerive, M. Bellomo, K. Belotskiy, O. Beltramello, N. L. Belyaev, O. Benary, D. Benchekroun, M. Bender, K. Bendtz, N. Benekos, Y. Benhammou, E. Benhar Noccioli, J. Benitez, J. A. Benitez Garcia, D. P. Benjamin, J. R. Bensinger, S. Bentvelsen, L. Beresford, M. Beretta, D. Berge, E. Bergeaas Kuutmann, N. Berger, J. Beringer, S. Berlendis, N. R. Bernard, C. Bernius, F. U. Bernlochner, T. Berry, P. Berta, C. Bertella, G. Bertoli, F. Bertolucci, I. A. Bertram, C. Bertsche, D. Bertsche, G. J. Besjes, O. Bessidskaia Bylund, M. Bessner, N. Besson, C. Betancourt, S. Bethke, A. J. Bevan, W. Bhimji, R. M. Bianchi, L. Bianchini, M. Bianco, O. Biebel, D. Biedermann, R. Bielski, N. V. Biesuz, M. Biglietti, J. Bilbao De Mendizabal, H. Bilokon, M. Bindi, S. Binet, A. Bingul, C. Bini, S. Biondi, D. M. Bjergaard, C. W. Black, J. E. Black, K. M. Black, D. Blackburn, R. E. Blair, J.-B. Blanchard, J. E. Blanco, T. Blazek, I. Bloch, C. Blocker, W. Blum, U. Blumenschein, S. Blunier, G. J. Bobbink, V. S. Bobrovnikov, S. S. Bocchetta, A. Bocci, C. Bock, M. Boehler, D. Boerner, J. A. Bogaerts, D. Bogavac, A. G. Bogdanchikov, C. Bohm, V. Boisvert, T. Bold, V. Boldea, A. S. Boldyrev, M. Bomben, M. Bona, M. Boonekamp, A. Borisov, G. Borissov, J. Bortfeldt, D. Bortoletto, V. Bortolotto, K. Bos, D. Boscherini, M. Bosman, J. D. Bossio Sola, J. Boudreau, J. Bouffard, E. V. Bouhova-Thacker, D. Boumediene, C. Bourdarios, S. K. Boutle, A. Boveia, J. Boyd, I. R. Boyko, J. Bracinik, A. Brandt, G. Brandt, O. Brandt, U. Bratzler, B. Brau, J. E. Brau, H. M. Braun, W. D. Breaden Madden, K. Brendlinger, A. J. Brennan, L. Brenner, R. Brenner, S. Bressler, T. M. Bristow, D. Britton, D. Britzger, F. M. Brochu, I. Brock, R. Brock, G. Brooijmans, T. Brooks, W. K. Brooks, J. Brosamer, E. Brost, J. H. Broughton, P. A. Bruckman de Renstrom, D. Bruncko, R. Bruneliere, A. Bruni, G. Bruni, BH Brunt, M. Bruschi, N. Bruscino, P. Bryant, L. Bryngemark, T. Buanes, Q. Buat, P. Buchholz, A. G. Buckley, I. A. Budagov, F. Buehrer, M. K. Bugge, O. Bulekov, D. Bullock, H. Burckhart, S. Burdin, C. D. Burgard, B. Burghgrave, K. Burka, S. Burke, I. Burmeister, E. Busato, D. Büscher, V. Büscher, P. Bussey, J. M. Butler, C. M. Buttar, J. M. Butterworth, P. Butti, W. Buttinger, A. Buzatu, A. R. Buzykaev, S. Cabrera Urbán, D. Caforio, V. M. Cairo, O. Cakir, N. Calace, P. Calafiura, A. Calandri, G. Calderini, P. Calfayan, L. P. Caloba, D. Calvet, S. Calvet, T. P. Calvet, R. Camacho Toro, S. Camarda, P. Camarri, D. Cameron, R. Caminal Armadans, C. Camincher, S. Campana, M. Campanelli, A. Camplani, A. Campoverde, V. Canale, A. Canepa, M. Cano Bret, J. Cantero, R. Cantrill, T. Cao, M. D. M. Capeans Garrido, I. Caprini, M. Caprini, M. Capua, R. Caputo, R. M. Carbone, R. Cardarelli, F. Cardillo, I. Carli, T. Carli, G. Carlino, L. Carminati, S. Caron, E. Carquin, G. D. Carrillo-Montoya, J. R. Carter, J. Carvalho, D. Casadei, M. P. Casado, M. Casolino, D. W. Casper, E. Castaneda-Miranda, A. Castelli, V. Castillo Gimenez, N. F. Castro, A. Catinaccio, J. R. Catmore, A. Cattai, J. Caudron, V. Cavaliere, E. Cavallaro, D. Cavalli, M. Cavalli-Sforza, V. Cavasinni, F. Ceradini, L. Cerda Alberich, B. C. Cerio, A. S. Cerqueira, A. Cerri, L. Cerrito, F. Cerutti, M. Cerv, A. Cervelli, S. A. Cetin, A. Chafaq, D. Chakraborty, S. K. Chan, Y. L. Chan, P. Chang, J. D. Chapman, D. G. Charlton, A. Chatterjee, C. C. Chau, C. A. Chavez Barajas, S. Che, S. Cheatham, A. Chegwidden, S. Chekanov, S. V. Chekulaev, G. A. Chelkov, M. A. Chelstowska, C. Chen, H. Chen, K. Chen, S. Chen, S. Chen, X. Chen, Y. Chen, H. C. Cheng, H. J. Cheng, Y. Cheng, A. Cheplakov, E. Cheremushkina, R. Cherkaoui El Moursli, V. Chernyatin, E. Cheu, L. Chevalier, V. Chiarella, G. Chiarelli, G. Chiodini, A. S. Chisholm, A. Chitan, M. V. Chizhov, K. Choi, A. R. Chomont, S. Chouridou, B. K. B. Chow, V. Christodoulou, D. Chromek-Burckhart, J. Chudoba, A. J. Chuinard, J. J. Chwastowski, L. Chytka, G. Ciapetti, A. K. Ciftci, D. Cinca, V. Cindro, I. A. Cioara, A. Ciocio, F. Cirotto, Z. H. Citron, M. Citterio, M. Ciubancan, A. Clark, B. L. Clark, M. R. Clark, P. J. Clark, R. N. Clarke, C. Clement, Y. Coadou, M. Cobal, A. Coccaro, J. Cochran, L. Coffey, L. Colasurdo, B. Cole, S. Cole, A. P. Colijn, J. Collot, T. Colombo, G. Compostella, P. Conde Muiño, E. Coniavitis, S. H. Connell, I. A. Connelly, V. Consorti, S. Constantinescu, C. Conta, G. Conti, F. Conventi, M. Cooke, B. D. Cooper, A. M. Cooper-Sarkar, K. J. R. Cormier, T. Cornelissen, M. Corradi, F. Corriveau, A. Corso-Radu, A. Cortes-Gonzalez, G. Cortiana, G. Costa, M. J. Costa, D. Costanzo, G. Cottin, G. Cowan, B. E. Cox, K. Cranmer, S. J. Crawley, G. Cree, S. Crépé-Renaudin, F. Crescioli, W. A. Cribbs, M. Crispin Ortuzar, M. Cristinziani, V. Croft, G. Crosetti, T. Cuhadar Donszelmann, J. Cummings, M. Curatolo, J. Cúth, C. Cuthbert, H. Czirr, P. Czodrowski, S. D’Auria, M. D’Onofrio, M. J. Da Cunha Sargedas De Sousa, C. Da Via, W. Dabrowski, T. Dado, T. Dai, O. Dale, F. Dallaire, C. Dallapiccola, M. Dam, J. R. Dandoy, N. P. Dang, A. C. Daniells, N. S. Dann, M. Danninger, M. Dano Hoffmann, V. Dao, G. Darbo, S. Darmora, J. Dassoulas, A. Dattagupta, W. Davey, C. David, T. Davidek, M. Davies, P. Davison, E. Dawe, I. Dawson, R. K. Daya-Ishmukhametova, K. De, R. de Asmundis, A. De Benedetti, S. De Castro, S. De Cecco, N. De Groot, P. de Jong, H. De la Torre, F. De Lorenzi, D. De Pedis, A. De Salvo, U. De Sanctis, A. De Santo, J. B. De Vivie De Regie, W. J. Dearnaley, R. Debbe, C. Debenedetti, D. V. Dedovich, I. Deigaard, M. Del Gaudio, J. Del Peso, T. Del Prete, D. Delgove, F. Deliot, C. M. Delitzsch, M. Deliyergiyev, A. Dell’Acqua, L. Dell’Asta, M. Dell’Orso, M. Della Pietra, D. della Volpe, M. Delmastro, P. A. Delsart, C. Deluca, D. A. DeMarco, S. Demers, M. Demichev, A. Demilly, S. P. Denisov, D. Denysiuk, D. Derendarz, J. E. Derkaoui, F. Derue, P. Dervan, K. Desch, C. Deterre, K. Dette, P. O. Deviveiros, A. Dewhurst, S. Dhaliwal, A. Di Ciaccio, L. Di Ciaccio, W. K. Di Clemente, C. Di Donato, A. Di Girolamo, B. Di Girolamo, B. Di Micco, R. Di Nardo, A. Di Simone, R. Di Sipio, D. Di Valentino, C. Diaconu, M. Diamond, F. A. Dias, M. A. Diaz, E. B. Diehl, J. Dietrich, S. Diglio, A. Dimitrievska, J. Dingfelder, P. Dita, S. Dita, F. Dittus, F. Djama, T. Djobava, J. I. Djuvsland, M. A. B. do Vale, D. Dobos, M. Dobre, C. Doglioni, T. Dohmae, J. Dolejsi, Z. Dolezal, B. A. Dolgoshein, M. Donadelli, S. Donati, P. Dondero, J. Donini, J. Dopke, A. Doria, M. T. Dova, A. T. Doyle, E. Drechsler, M. Dris, Y. Du, J. Duarte-Campderros, E. Duchovni, G. Duckeck, O. A. Ducu, D. Duda, A. Dudarev, L. Duflot, L. Duguid, M. Dührssen, M. Dumancic, M. Dunford, H. Duran Yildiz, M. Düren, A. Durglishvili, D. Duschinger, B. Dutta, M. Dyndal, C. Eckardt, K. M. Ecker, R. C. Edgar, N. C. Edwards, T. Eifert, G. Eigen, K. Einsweiler, T. Ekelof, M. El Kacimi, V. Ellajosyula, M. Ellert, S. Elles, F. Ellinghaus, A. A. Elliot, N. Ellis, J. Elmsheuser, M. Elsing, D. Emeliyanov, Y. Enari, O. C. Endner, M. Endo, J. S. Ennis, J. Erdmann, A. Ereditato, G. Ernis, J. Ernst, M. Ernst, S. Errede, E. Ertel, M. Escalier, H. Esch, C. Escobar, B. Esposito, A. I. Etienvre, E. Etzion, H. Evans, A. Ezhilov, F. Fabbri, L. Fabbri, G. Facini, R. M. Fakhrutdinov, S. Falciano, R. J. Falla, J. Faltova, Y. Fang, M. Fanti, A. Farbin, A. Farilla, C. Farina, T. Farooque, S. Farrell, S. M. Farrington, P. Farthouat, F. Fassi, P. Fassnacht, D. Fassouliotis, M. Faucci Giannelli, A. Favareto, W. J. Fawcett, L. Fayard, O. L. Fedin, W. Fedorko, S. Feigl, L. Feligioni, C. Feng, E. J. Feng, H. Feng, A. B. Fenyuk, L. Feremenga, P. Fernandez Martinez, S. Fernandez Perez, J. Ferrando, A. Ferrari, P. Ferrari, R. Ferrari, D. E. Ferreira de Lima, A. Ferrer, D. Ferrere, C. Ferretti, A. Ferretto Parodi, F. Fiedler, A. Filipčič, M. Filipuzzi, F. Filthaut, M. Fincke-Keeler, K. D. Finelli, M. C. N. Fiolhais, L. Fiorini, A. Firan, A. Fischer, C. Fischer, J. Fischer, W. C. Fisher, N. Flaschel, I. Fleck, P. Fleischmann, G. T. Fletcher, R. R. M. Fletcher, T. Flick, A. Floderus, L. R. Flores Castillo, M. J. Flowerdew, G. T. Forcolin, A. Formica, A. Forti, A. G. Foster, D. Fournier, H. Fox, S. Fracchia, P. Francavilla, M. Franchini, D. Francis, L. Franconi, M. Franklin, M. Frate, M. Fraternali, D. Freeborn, S. M. Fressard-Batraneanu, F. Friedrich, D. Froidevaux, J. A. Frost, C. Fukunaga, E. Fullana Torregrosa, T. Fusayasu, J. Fuster, C. Gabaldon, O. Gabizon, A. Gabrielli, A. Gabrielli, G. P. Gach, S. Gadatsch, S. Gadomski, G. Gagliardi, L. G. Gagnon, P. Gagnon, C. Galea, B. Galhardo, E. J. Gallas, B. J. Gallop, P. Gallus, G. Galster, K. K. Gan, J. Gao, Y. Gao, Y. S. Gao, F. M. Garay Walls, C. García, J. E. García Navarro, M. Garcia-Sciveres, R. W. Gardner, N. Garelli, V. Garonne, A. Gascon Bravo, C. Gatti, A. Gaudiello, G. Gaudio, B. Gaur, L. Gauthier, I. L. Gavrilenko, C. Gay, G. Gaycken, E. N. Gazis, Z. Gecse, C. N. P. Gee, Ch. Geich-Gimbel, M. P. Geisler, C. Gemme, M. H. Genest, C. Geng, S. Gentile, S. George, D. Gerbaudo, A. Gershon, S. Ghasemi, H. Ghazlane, M. Ghneimat, B. Giacobbe, S. Giagu, P. Giannetti, B. Gibbard, S. M. Gibson, M. Gignac, M. Gilchriese, T. P. S. Gillam, D. Gillberg, G. Gilles, D. M. Gingrich, N. Giokaris, M. P. Giordani, F. M. Giorgi, F. M. Giorgi, P. F. Giraud, P. Giromini, D. Giugni, F. Giuli, C. Giuliani, M. Giulini, B. K. Gjelsten, S. Gkaitatzis, I. Gkialas, E. L. Gkougkousis, L. K. Gladilin, C. Glasman, J. Glatzer, P. C. F. Glaysher, A. Glazov, M. Goblirsch-Kolb, J. Godlewski, S. Goldfarb, T. Golling, D. Golubkov, A. Gomes, R. Gonçalo, J. Goncalves Pinto Firmino Da Costa, L. Gonella, A. Gongadze, S. González de la Hoz, G. Gonzalez Parra, S. Gonzalez-Sevilla, L. Goossens, P. A. Gorbounov, H. A. Gordon, I. Gorelov, B. Gorini, E. Gorini, A. Gorišek, E. Gornicki, A. T. Goshaw, C. Gössling, M. I. Gostkin, C. R. Goudet, D. Goujdami, A. G. Goussiou, N. Govender, E. Gozani, L. Graber, I. Grabowska-Bold, P. O. J. Gradin, P. Grafström, J. Gramling, E. Gramstad, S. Grancagnolo, V. Gratchev, H. M. Gray, E. Graziani, Z. D. Greenwood, C. Grefe, K. Gregersen, I. M. Gregor, P. Grenier, K. Grevtsov, J. Griffiths, A. A. Grillo, K. Grimm, S. Grinstein, Ph. Gris, J.-F. Grivaz, S. Groh, J. P. Grohs, E. Gross, J. Grosse-Knetter, G. C. Grossi, Z. J. Grout, L. Guan, W. Guan, J. Guenther, F. Guescini, D. Guest, O. Gueta, E. Guido, T. Guillemin, S. Guindon, U. Gul, C. Gumpert, J. Guo, Y. Guo, S. Gupta, G. Gustavino, P. Gutierrez, N. G. Gutierrez Ortiz, C. Gutschow, C. Guyot, C. Gwenlan, C. B. Gwilliam, A. Haas, C. Haber, H. K. Hadavand, N. Haddad, A. Hadef, P. Haefner, S. Hageböck, Z. Hajduk, H. Hakobyan, M. Haleem, J. Haley, G. Halladjian, G. D. Hallewell, K. Hamacher, P. Hamal, K. Hamano, A. Hamilton, G. N. Hamity, P. G. Hamnett, L. Han, K. Hanagaki, K. Hanawa, M. Hance, B. Haney, P. Hanke, R. Hanna, J. B. Hansen, J. D. Hansen, M. C. Hansen, P. H. Hansen, K. Hara, A. S. Hard, T. Harenberg, F. Hariri, S. Harkusha, R. D. Harrington, P. F. Harrison, F. Hartjes, M. Hasegawa, Y. Hasegawa, A. Hasib, S. Hassani, S. Haug, R. Hauser, L. Hauswald, M. Havranek, C. M. Hawkes, R. J. Hawkings, A. D. Hawkins, D. Hayden, C. P. Hays, J. M. Hays, H. S. Hayward, S. J. Haywood, S. J. Head, T. Heck, V. Hedberg, L. Heelan, S. Heim, T. Heim, B. Heinemann, J. J. Heinrich, L. Heinrich, C. Heinz, J. Hejbal, L. Helary, S. Hellman, C. Helsens, J. Henderson, R. C. W. Henderson, Y. Heng, S. Henkelmann, A. M. Henriques Correia, S. Henrot-Versille, G. H. Herbert, H. Herde, Y. Hernández Jiménez, G. Herten, R. Hertenberger, L. Hervas, G. G. Hesketh, N. P. Hessey, J. W. Hetherly, R. Hickling, E. Higón-Rodriguez, E. Hill, J. C. Hill, K. H. Hiller, S. J. Hillier, I. Hinchliffe, E. Hines, R. R. Hinman, M. Hirose, D. Hirschbuehl, J. Hobbs, N. Hod, M. C. Hodgkinson, P. Hodgson, A. Hoecker, M. R. Hoeferkamp, F. Hoenig, M. Hohlfeld, D. Hohn, T. R. Holmes, M. Homann, T. M. Hong, B. H. Hooberman, W. H. Hopkins, Y. Horii, A. J. Horton, J-Y. Hostachy, S. Hou, A. Hoummada, J. Howarth, M. Hrabovsky, I. Hristova, J. Hrivnac, T. Hryn’ova, A. Hrynevich, C. Hsu, P. J. Hsu, S.-C. Hsu, D. Hu, Q. Hu, Y. Huang, Z. Hubacek, F. Hubaut, F. Huegging, T. B. Huffman, E. W. Hughes, G. Hughes, M. Huhtinen, T. A. Hülsing, P. Huo, N. Huseynov, J. Huston, J. Huth, G. Iacobucci, G. Iakovidis, I. Ibragimov, L. Iconomidou-Fayard, E. Ideal, Z. Idrissi, P. Iengo, O. Igonkina, T. Iizawa, Y. Ikegami, M. Ikeno, Y. Ilchenko, D. Iliadis, N. Ilic, T. Ince, G. Introzzi, P. Ioannou, M. Iodice, K. Iordanidou, V. Ippolito, M. Ishino, M. Ishitsuka, R. Ishmukhametov, C. Issever, S. Istin, F. Ito, J. M. Iturbe Ponce, R. Iuppa, W. Iwanski, H. Iwasaki, J. M. Izen, V. Izzo, S. Jabbar, B. Jackson, M. Jackson, P. Jackson, V. Jain, K. B. Jakobi, K. Jakobs, S. Jakobsen, T. Jakoubek, D. O. Jamin, D. K. Jana, E. Jansen, R. Jansky, J. Janssen, M. Janus, G. Jarlskog, N. Javadov, T. Javůrek, F. Jeanneau, L. Jeanty, J. Jejelava, G.-Y. Jeng, D. Jennens, P. Jenni, J. Jentzsch, C. Jeske, S. Jézéquel, H. Ji, J. Jia, H. Jiang, Y. Jiang, S. Jiggins, J. Jimenez Pena, S. Jin, A. Jinaru, O. Jinnouchi, P. Johansson, K. A. Johns, W. J. Johnson, K. Jon-And, G. Jones, R. W. L. Jones, S. Jones, T. J. Jones, J. Jongmanns, P. M. Jorge, J. Jovicevic, X. Ju, A. Juste Rozas, M. K. Köhler, A. Kaczmarska, M. Kado, H. Kagan, M. Kagan, S. J. Kahn, E. Kajomovitz, C. W. Kalderon, A. Kaluza, S. Kama, A. Kamenshchikov, N. Kanaya, S. Kaneti, L. Kanjir, V. A. Kantserov, J. Kanzaki, B. Kaplan, L. S. Kaplan, A. Kapliy, D. Kar, K. Karakostas, A. Karamaoun, N. Karastathis, M. J. Kareem, E. Karentzos, M. Karnevskiy, S. N. Karpov, Z. M. Karpova, K. Karthik, V. Kartvelishvili, A. N. Karyukhin, K. Kasahara, L. Kashif, R. D. Kass, A. Kastanas, Y. Kataoka, C. Kato, A. Katre, J. Katzy, K. Kawagoe, T. Kawamoto, G. Kawamura, S. Kazama, V. F. Kazanin, R. Keeler, R. Kehoe, J. S. Keller, J. J. Kempster, K Kentaro, H. Keoshkerian, O. Kepka, B. P. Kerševan, S. Kersten, R. A. Keyes, F. Khalil-zada, A. Khanov, A. G. Kharlamov, T. J. Khoo, V. Khovanskiy, E. Khramov, J. Khubua, S. Kido, H. Y. Kim, S. H. Kim, Y. K. Kim, N. Kimura, O. M. Kind, B. T. King, M. King, S. B. King, J. Kirk, A. E. Kiryunin, T. Kishimoto, D. Kisielewska, F. Kiss, K. Kiuchi, O. Kivernyk, E. Kladiva, M. H. Klein, M. Klein, U. Klein, K. Kleinknecht, P. Klimek, A. Klimentov, R. Klingenberg, J. A. Klinger, T. Klioutchnikova, E.-E. Kluge, P. Kluit, S. Kluth, J. Knapik, E. Kneringer, E. B. F. G. Knoops, A. Knue, A. Kobayashi, D. Kobayashi, T. Kobayashi, M. Kobel, M. Kocian, P. Kodys, N. M. Koehler, T. Koffas, E. Koffeman, T. Koi, H. Kolanoski, M. Kolb, I. Koletsou, A. A. Komar, Y. Komori, T. Kondo, N. Kondrashova, K. Köneke, A. C. König, T. Kono, R. Konoplich, N. Konstantinidis, R. Kopeliansky, S. Koperny, L. Köpke, A. K. Kopp, K. Korcyl, K. Kordas, A. Korn, A. A. Korol, I. Korolkov, E. V. Korolkova, O. Kortner, S. Kortner, T. Kosek, V. V. Kostyukhin, A. Kotwal, A. Kourkoumeli-Charalampidi, C. Kourkoumelis, V. Kouskoura, A. B. Kowalewska, R. Kowalewski, T. Z. Kowalski, W. Kozanecki, A. S. Kozhin, V. A. Kramarenko, G. Kramberger, D. Krasnopevtsev, M. W. Krasny, A. Krasznahorkay, J. K. Kraus, A. Kravchenko, M. Kretz, J. Kretzschmar, K. Kreutzfeldt, P. Krieger, K. Krizka, K. Kroeninger, H. Kroha, J. Kroll, J. Kroseberg, J. Krstic, U. Kruchonak, H. Krüger, N. Krumnack, A. Kruse, M. C. Kruse, M. Kruskal, T. Kubota, H. Kucuk, S. Kuday, J. T. Kuechler, S. Kuehn, A. Kugel, F. Kuger, A. Kuhl, T. Kuhl, V. Kukhtin, R. Kukla, Y. Kulchitsky, S. Kuleshov, M. Kuna, T. Kunigo, A. Kupco, H. Kurashige, Y. A. Kurochkin, V. Kus, E. S. Kuwertz, M. Kuze, J. Kvita, T. Kwan, D. Kyriazopoulos, A. La Rosa, J. L. La Rosa Navarro, L. La Rotonda, C. Lacasta, F. Lacava, J. Lacey, H. Lacker, D. Lacour, V. R. Lacuesta, E. Ladygin, R. Lafaye, B. Laforge, T. Lagouri, S. Lai, S. Lammers, W. Lampl, E. Lançon, U. Landgraf, M. P. J. Landon, V. S. Lang, J. C. Lange, A. J. Lankford, F. Lanni, K. Lantzsch, A. Lanza, S. Laplace, C. Lapoire, J. F. Laporte, T. Lari, F. Lasagni Manghi, M. Lassnig, P. Laurelli, W. Lavrijsen, A. T. Law, P. Laycock, T. Lazovich, M. Lazzaroni, B. Le, O. Le Dortz, E. Le Guirriec, E. Le Menedeu, E. P. Le Quilleuc, M. LeBlanc, T. LeCompte, F. Ledroit-Guillon, C. A. Lee, S. C. Lee, L. Lee, G. Lefebvre, M. Lefebvre, F. Legger, C. Leggett, A. Lehan, G. Lehmann Miotto, X. Lei, W. A. Leight, A. Leisos, A. G. Leister, M. A. L. Leite, R. Leitner, D. Lellouch, B. Lemmer, K. J. C. Leney, T. Lenz, B. Lenzi, R. Leone, S. Leone, C. Leonidopoulos, S. Leontsinis, G. Lerner, C. Leroy, A. A. J. Lesage, C. G. Lester, M. Levchenko, J. Levêque, D. Levin, L. J. Levinson, M. Levy, A. M. Leyko, M. Leyton, B. Li, H. Li, H. L. Li, L. Li, L. Li, Q. Li, S. Li, X. Li, Y. Li, Z. Liang, B. Liberti, A. Liblong, P. Lichard, K. Lie, J. Liebal, W. Liebig, A. Limosani, S. C. Lin, T. H. Lin, B. E. Lindquist, E. Lipeles, A. Lipniacka, M. Lisovyi, T. M. Liss, D. Lissauer, A. Lister, A. M. Litke, B. Liu, D. Liu, H. Liu, H. Liu, J. Liu, J. B. Liu, K. Liu, L. Liu, M. Liu, M. Liu, Y. L. Liu, Y. Liu, M. Livan, A. Lleres, J. Llorente Merino, S. L. Lloyd, F. Lo Sterzo, E. Lobodzinska, P. Loch, W. S. Lockman, F. K. Loebinger, A. E. Loevschall-Jensen, K. M. Loew, A. Loginov, T. Lohse, K. Lohwasser, M. Lokajicek, B. A. Long, J. D. Long, R. E. Long, L. Longo, K. A. Looper, L. Lopes, D. Lopez Mateos, B. Lopez Paredes, I. Lopez Paz, A. Lopez Solis, J. Lorenz, N. Lorenzo Martinez, M. Losada, P. J. Lösel, X. Lou, A. Lounis, J. Love, P. A. Love, H. Lu, N. Lu, H. J. Lubatti, C. Luci, A. Lucotte, C. Luedtke, F. Luehring, W. Lukas, L. Luminari, O. Lundberg, B. Lund-Jensen, D. Lynn, R. Lysak, E. Lytken, V. Lyubushkin, H. Ma, L. L. Ma, Y. Ma, G. Maccarrone, A. Macchiolo, C. M. Macdonald, B. Maček, J. Machado Miguens, D. Madaffari, R. Madar, H. J. Maddocks, W. F. Mader, A. Madsen, J. Maeda, S. Maeland, T. Maeno, A. Maevskiy, E. Magradze, J. Mahlstedt, C. Maiani, C. Maidantchik, A. A. Maier, T. Maier, A. Maio, S. Majewski, Y. Makida, N. Makovec, B. Malaescu, Pa. Malecki, V. P. Maleev, F. Malek, U. Mallik, D. Malon, C. Malone, S. Maltezos, S. Malyukov, J. Mamuzic, G. Mancini, B. Mandelli, L. Mandelli, I. Mandić, J. Maneira, L. Manhaes de Andrade Filho, J. Manjarres Ramos, A. Mann, B. Mansoulie, R. Mantifel, M. Mantoani, S. Manzoni, L. Mapelli, G. Marceca, L. March, G. Marchiori, M. Marcisovsky, M. Marjanovic, D. E. Marley, F. Marroquim, S. P. Marsden, Z. Marshall, S. Marti-Garcia, B. Martin, T. A. Martin, V. J. Martin, B. Martin dit Latour, M. Martinez, S. Martin-Haugh, V. S. Martoiu, A. C. Martyniuk, M. Marx, A. Marzin, L. Masetti, T. Mashimo, R. Mashinistov, J. Masik, A. L. Maslennikov, I. Massa, L. Massa, P. Mastrandrea, A. Mastroberardino, T. Masubuchi, P. Mättig, J. Mattmann, J. Maurer, S. J. Maxfield, D. A. Maximov, R. Mazini, S. M. Mazza, N. C. Mc Fadden, G. Mc Goldrick, S. P. Mc Kee, A. McCarn, R. L. McCarthy, T. G. McCarthy, L. I. McClymont, E. F. McDonald, K. W. McFarlane, J. A. Mcfayden, G. Mchedlidze, S. J. McMahon, R. A. McPherson, M. Medinnis, S. Meehan, S. Mehlhase, A. Mehta, K. Meier, C. Meineck, B. Meirose, B. R. Mellado Garcia, M. Melo, F. Meloni, A. Mengarelli, S. Menke, E. Meoni, S. Mergelmeyer, P. Mermod, L. Merola, C. Meroni, F. S. Merritt, A. Messina, J. Metcalfe, A. S. Mete, C. Meyer, C. Meyer, J-P. Meyer, J. Meyer, H. Meyer Zu Theenhausen, R. P. Middleton, S. Miglioranzi, L. Mijović, G. Mikenberg, M. Mikestikova, M. Mikuž, M. Milesi, A. Milic, D. W. Miller, C. Mills, A. Milov, D. A. Milstead, A. A. Minaenko, Y. Minami, I. A. Minashvili, A. I. Mincer, B. Mindur, M. Mineev, Y. Ming, L. M. Mir, K. P. Mistry, T. Mitani, J. Mitrevski, V. A. Mitsou, A. Miucci, P. S. Miyagawa, J. U. Mjörnmark, T. Moa, K. Mochizuki, S. Mohapatra, W. Mohr, S. Molander, R. Moles-Valls, R. Monden, M. C. Mondragon, K. Mönig, J. Monk, E. Monnier, A. Montalbano, J. Montejo Berlingen, F. Monticelli, S. Monzani, R. W. Moore, N. Morange, D. Moreno, M. Moreno Llácer, P. Morettini, D. Mori, T. Mori, M. Morii, M. Morinaga, V. Morisbak, S. Moritz, A. K. Morley, G. Mornacchi, J. D. Morris, S. S. Mortensen, L. Morvaj, M. Mosidze, J. Moss, K. Motohashi, R. Mount, E. Mountricha, S. V. Mouraviev, E. J. W. Moyse, S. Muanza, R. D. Mudd, F. Mueller, J. Mueller, R. S. P. Mueller, T. Mueller, D. Muenstermann, P. Mullen, G. A. Mullier, F. J. Munoz Sanchez, J. A. Murillo Quijada, W. J. Murray, H. Musheghyan, M. Muškinja, A. G. Myagkov, M. Myska, B. P. Nachman, O. Nackenhorst, J. Nadal, K. Nagai, R. Nagai, K. Nagano, Y. Nagasaka, K. Nagata, M. Nagel, E. Nagy, A. M. Nairz, Y. Nakahama, K. Nakamura, T. Nakamura, I. Nakano, H. Namasivayam, R. F. Naranjo Garcia, R. Narayan, D. I. Narrias Villar, I. Naryshkin, T. Naumann, G. Navarro, R. Nayyar, H. A. Neal, P. Yu. Nechaeva, T. J. Neep, P. D. Nef, A. Negri, M. Negrini, S. Nektarijevic, C. Nellist, A. Nelson, S. Nemecek, P. Nemethy, A. A. Nepomuceno, M. Nessi, M. S. Neubauer, M. Neumann, R. M. Neves, P. Nevski, P. R. Newman, D. H. Nguyen, T. Nguyen Manh, R. B. Nickerson, R. Nicolaidou, J. Nielsen, A. Nikiforov, V. Nikolaenko, I. Nikolic-Audit, K. Nikolopoulos, J. K. Nilsen, P. Nilsson, Y. Ninomiya, A. Nisati, R. Nisius, T. Nobe, L. Nodulman, M. Nomachi, I. Nomidis, T. Nooney, S. Norberg, M. Nordberg, N. Norjoharuddeen, O. Novgorodova, S. Nowak, M. Nozaki, L. Nozka, K. Ntekas, E. Nurse, F. Nuti, F. O’grady, D. C. O’Neil, A. A. O’Rourke, V. O’Shea, F. G. Oakham, H. Oberlack, T. Obermann, J. Ocariz, A. Ochi, I. Ochoa, J. P. Ochoa-Ricoux, S. Oda, S. Odaka, H. Ogren, A. Oh, S. H. Oh, C. C. Ohm, H. Ohman, H. Oide, H. Okawa, Y. Okumura, T. Okuyama, A. Olariu, L. F. Oleiro Seabra, S. A. Olivares Pino, D. Oliveira Damazio, A. Olszewski, J. Olszowska, A. Onofre, K. Onogi, P. U. E. Onyisi, M. J. Oreglia, Y. Oren, D. Orestano, N. Orlando, R. S. Orr, B. Osculati, R. Ospanov, G. Otero y Garzon, H. Otono, M. Ouchrif, F. Ould-Saada, A. Ouraou, K. P. Oussoren, Q. Ouyang, M. Owen, R. E. Owen, V. E. Ozcan, N. Ozturk, K. Pachal, A. Pacheco Pages, C. Padilla Aranda, M. Pagáčová, S. Pagan Griso, F. Paige, P. Pais, K. Pajchel, G. Palacino, S. Palestini, M. Palka, D. Pallin, A. Palma, E. St. Panagiotopoulou, C. E. Pandini, J. G. Panduro Vazquez, P. Pani, S. Panitkin, D. Pantea, L. Paolozzi, Th. D. Papadopoulou, K. Papageorgiou, A. Paramonov, D. Paredes Hernandez, A. J. Parker, M. A. Parker, K. A. Parker, F. Parodi, J. A. Parsons, U. Parzefall, V. R. Pascuzzi, E. Pasqualucci, S. Passaggio, F. Pastore, Fr. Pastore, G. Pásztor, S. Pataraia, J. R. Pater, T. Pauly, J. Pearce, B. Pearson, L. E. Pedersen, M. Pedersen, S. Pedraza Lopez, R. Pedro, S. V. Peleganchuk, D. Pelikan, O. Penc, C. Peng, H. Peng, J. Penwell, B. S. Peralva, M. M. Perego, D. V. Perepelitsa, E. Perez Codina, L. Perini, H. Pernegger, S. Perrella, R. Peschke, V. D. Peshekhonov, K. Peters, R. F. Y. Peters, B. A. Petersen, T. C. Petersen, E. Petit, A. Petridis, C. Petridou, P. Petroff, E. Petrolo, M. Petrov, F. Petrucci, N. E. Pettersson, A. Peyaud, R. Pezoa, P. W. Phillips, G. Piacquadio, E. Pianori, A. Picazio, E. Piccaro, M. Piccinini, M. A. Pickering, R. Piegaia, J. E. Pilcher, A. D. Pilkington, A. W. J. Pin, M. Pinamonti, J. L. Pinfold, A. Pingel, S. Pires, H. Pirumov, M. Pitt, L. Plazak, M.-A. Pleier, V. Pleskot, E. Plotnikova, P. Plucinski, D. Pluth, R. Poettgen, L. Poggioli, D. Pohl, G. Polesello, A. Poley, A. Policicchio, R. Polifka, A. Polini, C. S. Pollard, V. Polychronakos, K. Pommès, L. Pontecorvo, B. G. Pope, G. A. Popeneciu, D. S. Popovic, A. Poppleton, S. Pospisil, K. Potamianos, I. N. Potrap, C. J. Potter, C. T. Potter, G. Poulard, J. Poveda, V. Pozdnyakov, M. E. Pozo Astigarraga, P. Pralavorio, A. Pranko, S. Prell, D. Price, L. E. Price, M. Primavera, S. Prince, M. Proissl, K. Prokofiev, F. Prokoshin, S. Protopopescu, J. Proudfoot, M. Przybycien, D. Puddu, D. Puldon, M. Purohit, P. Puzo, J. Qian, G. Qin, Y. Qin, A. Quadt, W. B. Quayle, M. Queitsch-Maitland, D. Quilty, S. Raddum, V. Radeka, V. Radescu, S. K. Radhakrishnan, P. Radloff, P. Rados, F. Ragusa, G. Rahal, J. A. Raine, S. Rajagopalan, M. Rammensee, C. Rangel-Smith, M. G. Ratti, F. Rauscher, S. Rave, T. Ravenscroft, M. Raymond, A. L. Read, N. P. Readioff, D. M. Rebuzzi, A. Redelbach, G. Redlinger, R. Reece, K. Reeves, L. Rehnisch, J. Reichert, H. Reisin, C. Rembser, H. Ren, M. Rescigno, S. Resconi, O. L. Rezanova, P. Reznicek, R. Rezvani, R. Richter, S. Richter, E. Richter-Was, O. Ricken, M. Ridel, P. Rieck, C. J. Riegel, J. Rieger, O. Rifki, M. Rijssenbeek, A. Rimoldi, L. Rinaldi, B. Ristić, E. Ritsch, I. Riu, F. Rizatdinova, E. Rizvi, C. Rizzi, S. H. Robertson, A. Robichaud-Veronneau, D. Robinson, J. E. M. Robinson, A. Robson, C. Roda, Y. Rodina, A. Rodriguez Perez, D. Rodriguez Rodriguez, S. Roe, C. S. Rogan, O. Røhne, A. Romaniouk, M. Romano, S. M. Romano Saez, E. Romero Adam, N. Rompotis, M. Ronzani, L. Roos, E. Ros, S. Rosati, K. Rosbach, P. Rose, O. Rosenthal, V. Rossetti, E. Rossi, L. P. Rossi, J. H. N. Rosten, R. Rosten, M. Rotaru, I. Roth, J. Rothberg, D. Rousseau, C. R. Royon, A. Rozanov, Y. Rozen, X. Ruan, F. Rubbo, V. I. Rud, M. S. Rudolph, F. Rühr, A. Ruiz-Martinez, Z. Rurikova, N. A. Rusakovich, A. Ruschke, H. L. Russell, J. P. Rutherfoord, N. Ruthmann, Y. F. Ryabov, M. Rybar, G. Rybkin, S. Ryu, A. Ryzhov, G. F. Rzehorz, A. F. Saavedra, G. Sabato, S. Sacerdoti, H. F-W. Sadrozinski, R. Sadykov, F. Safai Tehrani, P. Saha, M. Sahinsoy, M. Saimpert, T. Saito, H. Sakamoto, Y. Sakurai, G. Salamanna, A. Salamon, J. E. Salazar Loyola, D. Salek, P. H. Sales De Bruin, D. Salihagic, A. Salnikov, J. Salt, D. Salvatore, F. Salvatore, A. Salvucci, A. Salzburger, D. Sammel, D. Sampsonidis, A. Sanchez, J. Sánchez, V. Sanchez Martinez, H. Sandaker, R. L. Sandbach, H. G. Sander, M. Sandhoff, C. Sandoval, R. Sandstroem, D. P. C. Sankey, M. Sannino, A. Sansoni, C. Santoni, R. Santonico, H. Santos, I. Santoyo Castillo, K. Sapp, A. Sapronov, J. G. Saraiva, B. Sarrazin, O. Sasaki, Y. Sasaki, K. Sato, G. Sauvage, E. Sauvan, G. Savage, P. Savard, C. Sawyer, L. Sawyer, J. Saxon, C. Sbarra, A. Sbrizzi, T. Scanlon, D. A. Scannicchio, M. Scarcella, V. Scarfone, J. Schaarschmidt, P. Schacht, D. Schaefer, R. Schaefer, J. Schaeffer, S. Schaepe, S. Schaetzel, U. Schäfer, A. C. Schaffer, D. Schaile, R. D. Schamberger, V. Scharf, V. A. Schegelsky, D. Scheirich, M. Schernau, C. Schiavi, C. Schillo, M. Schioppa, S. Schlenker, K. Schmieden, C. Schmitt, S. Schmitt, S. Schmitz, B. Schneider, U. Schnoor, L. Schoeffel, A. Schoening, B. D. Schoenrock, E. Schopf, A. L. S. Schorlemmer, M. Schott, J. Schovancova, S. Schramm, M. Schreyer, N. Schuh, M. J. Schultens, H.-C. Schultz-Coulon, H. Schulz, M. Schumacher, B. A. Schumm, Ph. Schune, C. Schwanenberger, A. Schwartzman, T. A. Schwarz, Ph. Schwegler, H. Schweiger, Ph. Schwemling, R. Schwienhorst, J. Schwindling, T. Schwindt, G. Sciolla, F. Scuri, F. Scutti, J. Searcy, P. Seema, S. C. Seidel, A. Seiden, F. Seifert, J. M. Seixas, G. Sekhniaidze, K. Sekhon, S. J. Sekula, D. M. Seliverstov, N. Semprini-Cesari, C. Serfon, L. Serin, L. Serkin, M. Sessa, R. Seuster, H. Severini, T. Sfiligoj, F. Sforza, A. Sfyrla, E. Shabalina, N. W. Shaikh, L. Y. Shan, R. Shang, J. T. Shank, M. Shapiro, P. B. Shatalov, K. Shaw, S. M. Shaw, A. Shcherbakova, C. Y. Shehu, P. Sherwood, L. Shi, S. Shimizu, C. O. Shimmin, M. Shimojima, M. Shiyakova, A. Shmeleva, D. Shoaleh Saadi, M. J. Shochet, S. Shojaii, S. Shrestha, E. Shulga, M. A. Shupe, P. Sicho, P. E. Sidebo, O. Sidiropoulou, D. Sidorov, A. Sidoti, F. Siegert, Dj. Sijacki, J. Silva, S. B. Silverstein, V. Simak, O. Simard, Lj. Simic, S. Simion, E. Simioni, B. Simmons, D. Simon, M. Simon, P. Sinervo, N. B. Sinev, M. Sioli, G. Siragusa, S. Yu. Sivoklokov, J. Sjölin, T. B. Sjursen, M. B. Skinner, H. P. Skottowe, P. Skubic, M. Slater, T. Slavicek, M. Slawinska, K. Sliwa, R. Slovak, V. Smakhtin, B. H. Smart, L. Smestad, S. Yu. Smirnov, Y. Smirnov, L. N. Smirnova, O. Smirnova, M. N. K. Smith, R. W. Smith, M. Smizanska, K. Smolek, A. A. Snesarev, S. Snyder, R. Sobie, F. Socher, A. Soffer, D. A. Soh, G. Sokhrannyi, C. A. Solans Sanchez, M. Solar, E. Yu. Soldatov, U. Soldevila, A. A. Solodkov, A. Soloshenko, O. V. Solovyanov, V. Solovyev, P. Sommer, H. Son, H. Y. Song, A. Sood, A. Sopczak, V. Sopko, V. Sorin, D. Sosa, C. L. Sotiropoulou, R. Soualah, A. M. Soukharev, D. South, B. C. Sowden, S. Spagnolo, M. Spalla, M. Spangenberg, F. Spanò, D. Sperlich, F. Spettel, R. Spighi, G. Spigo, L. A. Spiller, M. Spousta, R. D. St. Denis, A. Stabile, R. Stamen, S. Stamm, E. Stanecka, R. W. Stanek, C. Stanescu, M. Stanescu-Bellu, M. M. Stanitzki, S. Stapnes, E. A. Starchenko, G. H. Stark, J. Stark, P. Staroba, P. Starovoitov, S. Stärz, R. Staszewski, P. Steinberg, B. Stelzer, H. J. Stelzer, O. Stelzer-Chilton, H. Stenzel, G. A. Stewart, J. A. Stillings, M. C. Stockton, M. Stoebe, G. Stoicea, P. Stolte, S. Stonjek, A. R. Stradling, A. Straessner, M. E. Stramaglia, J. Strandberg, S. Strandberg, A. Strandlie, M. Strauss, P. Strizenec, R. Ströhmer, D. M. Strom, R. Stroynowski, A. Strubig, S. A. Stucci, B. Stugu, N. A. Styles, D. Su, J. Su, R. Subramaniam, S. Suchek, Y. Sugaya, M. Suk, V. V. Sulin, S. Sultansoy, T. Sumida, S. Sun, X. Sun, J. E. Sundermann, K. Suruliz, G. Susinno, M. R. Sutton, S. Suzuki, M. Svatos, M. Swiatlowski, I. Sykora, T. Sykora, D. Ta, C. Taccini, K. Tackmann, J. Taenzer, A. Taffard, R. Tafirout, N. Taiblum, H. Takai, R. Takashima, T. Takeshita, Y. Takubo, M. Talby, A. A. Talyshev, J. Y. C. Tam, K. G. Tan, J. Tanaka, R. Tanaka, S. Tanaka, B. B. Tannenwald, S. Tapia Araya, S. Tapprogge, S. Tarem, G. F. Tartarelli, P. Tas, M. Tasevsky, T. Tashiro, E. Tassi, A. Tavares Delgado, Y. Tayalati, A. C. Taylor, G. N. Taylor, P. T. E. Taylor, W. Taylor, F. A. Teischinger, P. Teixeira-Dias, K. K. Temming, D. Temple, H. Ten Kate, P. K. Teng, J. J. Teoh, F. Tepel, S. Terada, K. Terashi, J. Terron, S. Terzo, M. Testa, R. J. Teuscher, T. Theveneaux-Pelzer, J. P. Thomas, J. Thomas-Wilsker, E. N. Thompson, P. D. Thompson, A. S. Thompson, L. A. Thomsen, E. Thomson, M. Thomson, M. J. Tibbetts, R. E. Ticse Torres, V. O. Tikhomirov, Yu. A. Tikhonov, S. Timoshenko, P. Tipton, S. Tisserant, K. Todome, T. Todorov, S. Todorova-Nova, J. Tojo, S. Tokár, K. Tokushuku, E. Tolley, L. Tomlinson, M. Tomoto, L. Tompkins, K. Toms, B. Tong, E. Torrence, H. Torres, E. Torró Pastor, J. Toth, F. Touchard, D. R. Tovey, T. Trefzger, A. Tricoli, I. M. Trigger, S. Trincaz-Duvoid, M. F. Tripiana, W. Trischuk, B. Trocmé, A. Trofymov, C. Troncon, M. Trottier-McDonald, M. Trovatelli, L. Truong, M. Trzebinski, A. Trzupek, J. C-L. Tseng, P. V. Tsiareshka, G. Tsipolitis, N. Tsirintanis, S. Tsiskaridze, V. Tsiskaridze, E. G. Tskhadadze, K. M. Tsui, I. I. Tsukerman, V. Tsulaia, S. Tsuno, D. Tsybychev, A. Tudorache, V. Tudorache, A. N. Tuna, S. A. Tupputi, S. Turchikhin, D. Turecek, D. Turgeman, R. Turra, A. J. Turvey, P. M. Tuts, M. Tyndel, G. Ucchielli, I. Ueda, R. Ueno, M. Ughetto, F. Ukegawa, G. Unal, A. Undrus, G. Unel, F. C. Ungaro, Y. Unno, C. Unverdorben, J. Urban, P. Urquijo, P. Urrejola, G. Usai, A. Usanova, L. Vacavant, V. Vacek, B. Vachon, C. Valderanis, E. Valdes Santurio, N. Valencic, S. Valentinetti, A. Valero, L. Valery, S. Valkar, S. Vallecorsa, J. A. Valls Ferrer, W. Van Den Wollenberg, P. C. Van Der Deijl, R. van der Geer, H. van der Graaf, N. van Eldik, P. van Gemmeren, J. Van Nieuwkoop, I. van Vulpen, M. C. van Woerden, M. Vanadia, W. Vandelli, R. Vanguri, A. Vaniachine, P. Vankov, G. Vardanyan, R. Vari, E. W. Varnes, T. Varol, D. Varouchas, A. Vartapetian, K. E. Varvell, J. G. Vasquez, F. Vazeille, T. Vazquez Schroeder, J. Veatch, L. M. Veloce, F. Veloso, S. Veneziano, A. Ventura, M. Venturi, N. Venturi, A. Venturini, V. Vercesi, M. Verducci, W. Verkerke, J. C. Vermeulen, A. Vest, M. C. Vetterli, O. Viazlo, I. Vichou, T. Vickey, O. E. Vickey Boeriu, G. H. A. Viehhauser, S. Viel, L. Vigani, R. Vigne, M. Villa, M. Villaplana Perez, E. Vilucchi, M. G. Vincter, V. B. Vinogradov, C. Vittori, I. Vivarelli, S. Vlachos, M. Vlasak, M. Vogel, P. Vokac, G. Volpi, M. Volpi, H. von der Schmitt, E. von Toerne, V. Vorobel, K. Vorobev, M. Vos, R. Voss, J. H. Vossebeld, N. Vranjes, M. Vranjes Milosavljevic, V. Vrba, M. Vreeswijk, R. Vuillermet, I. Vukotic, Z. Vykydal, P. Wagner, W. Wagner, H. Wahlberg, S. Wahrmund, J. Wakabayashi, J. Walder, R. Walker, W. Walkowiak, V. Wallangen, C. Wang, C. Wang, F. Wang, H. Wang, H. Wang, J. Wang, J. Wang, K. Wang, R. Wang, S. M. Wang, T. Wang, T. Wang, X. Wang, C. Wanotayaroj, A. Warburton, C. P. Ward, D. R. Wardrope, A. Washbrook, P. M. Watkins, A. T. Watson, M. F. Watson, G. Watts, S. Watts, B. M. Waugh, S. Webb, M. S. Weber, S. W. Weber, J. S. Webster, A. R. Weidberg, B. Weinert, J. Weingarten, C. Weiser, H. Weits, P. S. Wells, T. Wenaus, T. Wengler, S. Wenig, N. Wermes, M. Werner, P. Werner, M. Wessels, J. Wetter, K. Whalen, N. L. Whallon, A. M. Wharton, A. White, M. J. White, R. White, S. White, D. Whiteson, F. J. Wickens, W. Wiedenmann, M. Wielers, P. Wienemann, C. Wiglesworth, L. A. M. Wiik-Fuchs, A. Wildauer, F. Wilk, H. G. Wilkens, H. H. Williams, S. Williams, C. Willis, S. Willocq, J. A. Wilson, I. Wingerter-Seez, F. Winklmeier, O. J. Winston, B. T. Winter, M. Wittgen, J. Wittkowski, S. J. Wollstadt, M. W. Wolter, H. Wolters, B. K. Wosiek, J. Wotschack, M. J. Woudstra, K. W. Wozniak, M. Wu, M. Wu, S. L. Wu, X. Wu, Y. Wu, T. R. Wyatt, B. M. Wynne, S. Xella, D. Xu, L. Xu, B. Yabsley, S. Yacoob, R. Yakabe, D. Yamaguchi, Y. Yamaguchi, A. Yamamoto, S. Yamamoto, T. Yamanaka, K. Yamauchi, Y. Yamazaki, Z. Yan, H. Yang, H. Yang, Y. Yang, Z. Yang, W-M. Yao, Y. C. Yap, Y. Yasu, E. Yatsenko, K. H. Yau Wong, J. Ye, S. Ye, I. Yeletskikh, A. L. Yen, E. Yildirim, K. Yorita, R. Yoshida, K. Yoshihara, C. Young, C. J. S. Young, S. Youssef, D. R. Yu, J. Yu, J. M. Yu, J. Yu, L. Yuan, S. P. Y. Yuen, I. Yusuff, B. Zabinski, R. Zaidan, A. M. Zaitsev, N. Zakharchuk, J. Zalieckas, A. Zaman, S. Zambito, L. Zanello, D. Zanzi, C. Zeitnitz, M. Zeman, A. Zemla, J. C. Zeng, Q. Zeng, K. Zengel, O. Zenin, T. Ženiš, D. Zerwas, D. Zhang, F. Zhang, G. Zhang, H. Zhang, J. Zhang, L. Zhang, R. Zhang, R. Zhang, X. Zhang, Z. Zhang, X. Zhao, Y. Zhao, Z. Zhao, A. Zhemchugov, J. Zhong, B. Zhou, C. Zhou, L. Zhou, L. Zhou, M. Zhou, N. Zhou, C. G. Zhu, H. Zhu, J. Zhu, Y. Zhu, X. Zhuang, K. Zhukov, A. Zibell, D. Zieminska, N. I. Zimine, C. Zimmermann, S. Zimmermann, Z. Zinonos, M. Zinser, M. Ziolkowski, L. Živković, G. Zobernig, A. Zoccoli, M. zur Nedden, G. Zurzolo, L. Zwalinski

**Affiliations:** 1 Department of Physics, University of Adelaide, Adelaide, Australia; 2 Physics Department, SUNY Albany, Albany, NY USA; 3 Department of Physics, University of Alberta, Edmonton, AB Canada; 4 Department of Physics, Ankara University, Ankara, Turkey; 5 Istanbul Aydin University, Istanbul, Turkey; 6 Division of Physics, TOBB University of Economics and Technology, Ankara, Turkey; 7 LAPP, CNRS/IN2P3 and Université Savoie Mont Blanc, Annecy-le-Vieux, France; 8 High Energy Physics Division, Argonne National Laboratory, Argonne, IL USA; 9 Department of Physics, University of Arizona, Tucson, AZ USA; 10 Department of Physics, The University of Texas at Arlington, Arlington, TX USA; 11 Physics Department, University of Athens, Athens, Greece; 12 Physics Department, National Technical University of Athens, Zografou, Greece; 13 Department of Physics, The University of Texas at Austin, Austin, TX USA; 14 Institute of Physics, Azerbaijan Academy of Sciences, Baku, Azerbaijan; 15 Institut de Física d’Altes Energies (IFAE), The Barcelona Institute of Science and Technology, Barcelona, Spain; 16 Institute of Physics, University of Belgrade, Belgrade, Serbia; 17 Department for Physics and Technology, University of Bergen, Bergen, Norway; 18 Physics Division, Lawrence Berkeley National Laboratory and University of California, Berkeley, CA USA; 19 Department of Physics, Humboldt University, Berlin, Germany; 20 Albert Einstein Center for Fundamental Physics and Laboratory for High Energy Physics, University of Bern, Bern, Switzerland; 21 School of Physics and Astronomy, University of Birmingham, Birmingham, UK; 22 Department of Physics, Bogazici University, Istanbul, Turkey; 23 Department of Physics Engineering, Gaziantep University, Gaziantep, Turkey; 24 Faculty of Engineering and Natural Sciences, Istanbul Bilgi University, Istanbul, Turkey; 25 Faculty of Engineering and Natural Sciences, Bahcesehir University, Istanbul, Turkey; 26 Centro de Investigaciones, Universidad Antonio Narino, Bogota, Colombia; 27 INFN Sezione di Bologna, Bologna, Italy; 28 Dipartimento di Fisica e Astronomia, Università di Bologna, Bologna, Italy; 29 Physikalisches Institut, University of Bonn, Bonn, Germany; 30 Department of Physics, Boston University, Boston, MA USA; 31 Department of Physics, Brandeis University, Waltham, MA USA; 32 Universidade Federal do Rio De Janeiro COPPE/EE/IF, Rio de Janeiro, Brazil; 33 Electrical Circuits Department, Federal University of Juiz de Fora (UFJF), Juiz de Fora, Brazil; 34 Federal University of Sao Joao del Rei (UFSJ), Sao Joao del Rei, Brazil; 35 Instituto de Fisica, Universidade de Sao Paulo, São Paulo, Brazil; 36 Physics Department, Brookhaven National Laboratory, Upton, NY USA; 37 Transilvania University of Brasov, Brasov, Romania; 38 National Institute of Physics and Nuclear Engineering, Bucharest, Romania; 39 Physics Department, National Institute for Research and Development of Isotopic and Molecular Technologies, Cluj Napoca, Romania; 40 University Politehnica Bucharest, Bucharest, Romania; 41 West University in Timisoara, Timisoara, Romania; 42 Departamento de Física, Universidad de Buenos Aires, Buenos Aires, Argentina; 43 Cavendish Laboratory, University of Cambridge, Cambridge, UK; 44 Department of Physics, Carleton University, Ottawa, ON Canada; 45 CERN, Geneva, Switzerland; 46 Enrico Fermi Institute, University of Chicago, Chicago, IL USA; 47 Departamento de Física, Pontificia Universidad Católica de Chile, Santiago, Chile; 48 Departamento de Física, Universidad Técnica Federico Santa María, Valparaiso, Chile; 49 Institute of High Energy Physics, Chinese Academy of Sciences, Beijing, China; 50 Department of Modern Physics, University of Science and Technology of China, Hefei, Anhui China; 51 Department of Physics, Nanjing University, Nanjing, Jiangsu China; 52 School of Physics, Shandong University, Jinan, Shandong China; 53 Shanghai Key Laboratory for Particle Physics and Cosmology, Department of Physics and Astronomy, Shanghai Jiao Tong University (also affiliated with PKU-CHEP), Shanghai, China; 54 Physics Department, Tsinghua University, Beijing, 100084 China; 55 Laboratoire de Physique Corpusculaire, Clermont Université and Université Blaise Pascal and CNRS/IN2P3, Clermont-Ferrand, France; 56 Nevis Laboratory, Columbia University, Irvington, NY USA; 57 Niels Bohr Institute, University of Copenhagen, Kobenhavn, Denmark; 58 INFN Gruppo Collegato di Cosenza, Laboratori Nazionali di Frascati, Frascati, Italy; 59 Dipartimento di Fisica, Università della Calabria, Rende, Italy; 60 Faculty of Physics and Applied Computer Science, AGH University of Science and Technology, Kraków, Poland; 61 Marian Smoluchowski Institute of Physics, Jagiellonian University, Kraków, Poland; 62 Institute of Nuclear Physics, Polish Academy of Sciences, Krakow, Poland; 63 Physics Department, Southern Methodist University, Dallas, TX USA; 64 Physics Department, University of Texas at Dallas, Richardson, TX USA; 65 DESY, Hamburg and Zeuthen, Germany; 66 Institut für Experimentelle Physik IV, Technische Universität Dortmund, Dortmund, Germany; 67 Institut für Kern- und Teilchenphysik, Technische Universität Dresden, Dresden, Germany; 68 Department of Physics, Duke University, Durham, NC USA; 69 SUPA-School of Physics and Astronomy, University of Edinburgh, Edinburgh, UK; 70 INFN Laboratori Nazionali di Frascati, Frascati, Italy; 71 Fakultät für Mathematik und Physik, Albert-Ludwigs-Universität, Freiburg, Germany; 72 Section de Physique, Université de Genève, Geneva, Switzerland; 73 INFN Sezione di Genova, Genoa, Italy; 74 Dipartimento di Fisica, Università di Genova, Genoa, Italy; 75 E. Andronikashvili Institute of Physics, Iv. Javakhishvili Tbilisi State University, Tbilisi, Georgia; 76 High Energy Physics Institute, Tbilisi State University, Tbilisi, Georgia; 77 II Physikalisches Institut, Justus-Liebig-Universität Giessen, Giessen, Germany; 78 SUPA-School of Physics and Astronomy, University of Glasgow, Glasgow, UK; 79 II Physikalisches Institut, Georg-August-Universität, Göttingen, Germany; 80 Laboratoire de Physique Subatomique et de Cosmologie, Université Grenoble-Alpes, CNRS/IN2P3, Grenoble, France; 81 Department of Physics, Hampton University, Hampton, VA USA; 82 Laboratory for Particle Physics and Cosmology, Harvard University, Cambridge, MA USA; 83 Kirchhoff-Institut für Physik, Ruprecht-Karls-Universität Heidelberg, Heidelberg, Germany; 84 Physikalisches Institut, Ruprecht-Karls-Universität Heidelberg, Heidelberg, Germany; 85 ZITI Institut für technische Informatik, Ruprecht-Karls-Universität Heidelberg, Mannheim, Germany; 86 Faculty of Applied Information Science, Hiroshima Institute of Technology, Hiroshima, Japan; 87 Department of Physics, The Chinese University of Hong Kong, Shatin, NT Hong Kong; 88 Department of Physics, The University of Hong Kong, Pokfulam, Hong Kong; 89 Department of Physics, The Hong Kong University of Science and Technology, Clear Water Bay, Kowloon, Hong Kong, China; 90 Department of Physics, Indiana University, Bloomington, IN USA; 91 Institut für Astro- und Teilchenphysik, Leopold-Franzens-Universität, Innsbruck, Austria; 92 University of Iowa, Iowa City, IA USA; 93 Department of Physics and Astronomy, Iowa State University, Ames, IA USA; 94 Joint Institute for Nuclear Research, JINR Dubna, Dubna, Russia; 95 KEK, High Energy Accelerator Research Organization, Tsukuba, Japan; 96 Graduate School of Science, Kobe University, Kobe, Japan; 97 Faculty of Science, Kyoto University, Kyoto, Japan; 98 Kyoto University of Education, Kyoto, Japan; 99 Department of Physics, Kyushu University, Fukuoka, Japan; 100 Instituto de Física La Plata, Universidad Nacional de La Plata and CONICET, La Plata, Argentina; 101 Physics Department, Lancaster University, Lancaster, UK; 102 INFN Sezione di Lecce, Lecce, Italy; 103 Dipartimento di Matematica e Fisica, Università del Salento, Lecce, Italy; 104 Oliver Lodge Laboratory, University of Liverpool, Liverpool, UK; 105 Department of Physics, Jožef Stefan Institute and University of Ljubljana, Ljubljana, Slovenia; 106 School of Physics and Astronomy, Queen Mary University of London, London, UK; 107 Department of Physics, Royal Holloway University of London, Surrey, UK; 108 Department of Physics and Astronomy, University College London, London, UK; 109 Louisiana Tech University, Ruston, LA USA; 110 Laboratoire de Physique Nucléaire et de Hautes Energies, UPMC and Université Paris-Diderot and CNRS/IN2P3, Paris, France; 111 Fysiska institutionen, Lunds universitet, Lund, Sweden; 112 Departamento de Fisica Teorica C-15, Universidad Autonoma de Madrid, Madrid, Spain; 113 Institut für Physik, Universität Mainz, Mainz, Germany; 114 School of Physics and Astronomy, University of Manchester, Manchester, UK; 115 CPPM, Aix-Marseille Université and CNRS/IN2P3, Marseille, France; 116 Department of Physics, University of Massachusetts, Amherst, MA USA; 117 Department of Physics, McGill University, Montreal, QC Canada; 118 School of Physics, University of Melbourne, Melbourne, VIC Australia; 119 Department of Physics, The University of Michigan, Ann Arbor, MI USA; 120 Department of Physics and Astronomy, Michigan State University, East Lansing, MI USA; 121 INFN Sezione di Milano, Milan, Italy; 122 Dipartimento di Fisica, Università di Milano, Milan, Italy; 123 B.I. Stepanov Institute of Physics, National Academy of Sciences of Belarus, Minsk, Republic of Belarus; 124 National Scientific and Educational Centre for Particle and High Energy Physics, Minsk, Republic of Belarus; 125 Group of Particle Physics, University of Montreal, Montreal, QC Canada; 126 P.N. Lebedev Physical Institute of the Russian, Academy of Sciences, Moscow, Russia; 127 Institute for Theoretical and Experimental Physics (ITEP), Moscow, Russia; 128 National Research Nuclear University MEPhI, Moscow, Russia; 129 D.V. Skobeltsyn Institute of Nuclear Physics, M.V. Lomonosov Moscow State University, Moscow, Russia; 130 Fakultät für Physik, Ludwig-Maximilians-Universität München, Munich, Germany; 131 Max-Planck-Institut für Physik (Werner-Heisenberg-Institut), Munich, Germany; 132 Nagasaki Institute of Applied Science, Nagasaki, Japan; 133 Graduate School of Science and Kobayashi-Maskawa Institute, Nagoya University, Nagoya, Japan; 134 INFN Sezione di Napoli, Naples, Italy; 135 Dipartimento di Fisica, Università di Napoli, Naples, Italy; 136 Department of Physics and Astronomy, University of New Mexico, Albuquerque, NM USA; 137 Institute for Mathematics, Astrophysics and Particle Physics, Radboud University Nijmegen/Nikhef, Nijmegen, The Netherlands; 138 Nikhef National Institute for Subatomic Physics and University of Amsterdam, Amsterdam, The Netherlands; 139 Department of Physics, Northern Illinois University, DeKalb, IL USA; 140 Budker Institute of Nuclear Physics, SB RAS, Novosibirsk, Russia; 141 Department of Physics, New York University, New York, NY USA; 142 Ohio State University, Columbus, OH USA; 143 Faculty of Science, Okayama University, Okayama, Japan; 144 Homer L. Dodge Department of Physics and Astronomy, University of Oklahoma, Norman, OK USA; 145 Department of Physics, Oklahoma State University, Stillwater, OK USA; 146 Palacký University, RCPTM, Olomouc, Czech Republic; 147 Center for High Energy Physics, University of Oregon, Eugene, OR USA; 148 LAL, Univ. Paris-Sud, CNRS/IN2P3, Université Paris Saclay, Orsay, France; 149 Graduate School of Science, Osaka University, Osaka, Japan; 150 Department of Physics, University of Oslo, Oslo, Norway; 151 Department of Physics, Oxford University, Oxford, UK; 152 INFN Sezione di Pavia, Pavia, Italy; 153 Dipartimento di Fisica, Università di Pavia, Pavia, Italy; 154 Department of Physics, University of Pennsylvania, Philadelphia, PA USA; 155 National Research Centre “Kurchatov Institute” B.P.Konstantinov Petersburg Nuclear Physics Institute, St. Petersburg, Russia; 156 INFN Sezione di Pisa, Pisa, Italy; 157 Dipartimento di Fisica E. Fermi, Università di Pisa, Pisa, Italy; 158 Department of Physics and Astronomy, University of Pittsburgh, Pittsburgh, PA USA; 159 Laboratório de Instrumentação e Física Experimental de Partículas-LIP, Lisbon, Portugal; 160 Faculdade de Ciências, Universidade de Lisboa, Lisbon, Portugal; 161 Department of Physics, University of Coimbra, Coimbra, Portugal; 162 Centro de Física Nuclear da Universidade de Lisboa, Lisbon, Portugal; 163 Departamento de Fisica, Universidade do Minho, Braga, Portugal; 164 Departamento de Fisica Teorica y del Cosmos and CAFPE, Universidad de Granada, Granada, Spain; 165 Dep Fisica and CEFITEC of Faculdade de Ciencias e Tecnologia, Universidade Nova de Lisboa, Caparica, Portugal; 166 Institute of Physics, Academy of Sciences of the Czech Republic, Prague, Czech Republic; 167 Czech Technical University in Prague, Prague, Czech Republic; 168 Faculty of Mathematics and Physics, Charles University in Prague, Prague, Czech Republic; 169 State Research Center Institute for High Energy Physics, Protvino, NRC KI Russia; 170 Particle Physics Department, Rutherford Appleton Laboratory, Didcot, UK; 171 INFN Sezione di Roma, Rome, Italy; 172 Dipartimento di Fisica, Sapienza Università di Roma, Rome, Italy; 173 INFN Sezione di Roma Tor Vergata, Rome, Italy; 174 Dipartimento di Fisica, Università di Roma Tor Vergata, Rome, Italy; 175 INFN Sezione di Roma Tre, Rome, Italy; 176 Dipartimento di Matematica e Fisica, Università Roma Tre, Rome, Italy; 177 Faculté des Sciences Ain Chock, Réseau Universitaire de Physique des Hautes Energies-Université Hassan II, Casablanca, Morocco; 178 Centre National de l’Energie des Sciences Techniques Nucleaires, Rabat, Morocco; 179 Faculté des Sciences Semlalia, Université Cadi Ayyad, LPHEA-Marrakech, Marrakech, Morocco; 180 Faculté des Sciences, Université Mohamed Premier and LPTPM, Oujda, Morocco; 181 Faculté des Sciences, Université Mohammed V, Rabat, Morocco; 182 DSM/IRFU (Institut de Recherches sur les Lois Fondamentales de l’Univers), CEA Saclay (Commissariat à l’Energie Atomique et aux Energies Alternatives), Gif-sur-Yvette, France; 183 Santa Cruz Institute for Particle Physics, University of California Santa Cruz, Santa Cruz, CA USA; 184 Department of Physics, University of Washington, Seattle, WA USA; 185 Department of Physics and Astronomy, University of Sheffield, Sheffield, UK; 186 Department of Physics, Shinshu University, Nagano, Japan; 187 Fachbereich Physik, Universität Siegen, Siegen, Germany; 188 Department of Physics, Simon Fraser University, Burnaby, BC Canada; 189 SLAC National Accelerator Laboratory, Stanford, CA USA; 190 Faculty of Mathematics, Physics and Informatics, Comenius University, Bratislava, Slovak Republic; 191 Department of Subnuclear Physics, Institute of Experimental Physics of the Slovak Academy of Sciences, Kosice, Slovak Republic; 192 Department of Physics, University of Cape Town, Cape Town, South Africa; 193 Department of Physics, University of Johannesburg, Johannesburg, South Africa; 194 School of Physics, University of the Witwatersrand, Johannesburg, South Africa; 195 Department of Physics, Stockholm University, Stockholm, Sweden; 196 The Oskar Klein Centre, Stockholm, Sweden; 197 Physics Department, Royal Institute of Technology, Stockholm, Sweden; 198 Departments of Physics and Astronomy and Chemistry, Stony Brook University, Stony Brook, NY USA; 199 Department of Physics and Astronomy, University of Sussex, Brighton, UK; 200 School of Physics, University of Sydney, Sydney, Australia; 201 Institute of Physics, Academia Sinica, Taipei, Taiwan; 202 Department of Physics, Technion: Israel Institute of Technology, Haifa, Israel; 203 Raymond and Beverly Sackler School of Physics and Astronomy, Tel Aviv University, Tel Aviv, Israel; 204 Department of Physics, Aristotle University of Thessaloniki, Thessaloníki, Greece; 205 International Center for Elementary Particle Physics and Department of Physics, The University of Tokyo, Tokyo, Japan; 206 Graduate School of Science and Technology, Tokyo Metropolitan University, Tokyo, Japan; 207 Department of Physics, Tokyo Institute of Technology, Tokyo, Japan; 208 Department of Physics, University of Toronto, Toronto, ON Canada; 209 TRIUMF, Vancouver, BC Canada; 210 Department of Physics and Astronomy, York University, Toronto, ON Canada; 211 Faculty of Pure and Applied Sciences, and Center for Integrated Research in Fundamental Science and Engineering, University of Tsukuba, Tsukuba, Japan; 212 Department of Physics and Astronomy, Tufts University, Medford, MA USA; 213 Department of Physics and Astronomy, University of California Irvine, Irvine, CA USA; 214 INFN Gruppo Collegato di Udine, Sezione di Trieste, Udine, Italy; 215 ICTP, Trieste, Italy; 216 Dipartimento di Chimica, Fisica e Ambiente, Università di Udine, Udine, Italy; 217 Department of Physics and Astronomy, University of Uppsala, Uppsala, Sweden; 218 Department of Physics, University of Illinois, Urbana, IL USA; 219 Instituto de Fisica Corpuscular (IFIC) and Departamento de Fisica Atomica, Molecular y Nuclear and Departamento de Ingeniería Electrónica and Instituto de Microelectrónica de Barcelona (IMB-CNM), University of Valencia and CSIC, Valencia, Spain; 220 Department of Physics, University of British Columbia, Vancouver, BC Canada; 221 Department of Physics and Astronomy, University of Victoria, Victoria, BC Canada; 222 Department of Physics, University of Warwick, Coventry, UK; 223 Waseda University, Tokyo, Japan; 224 Department of Particle Physics, The Weizmann Institute of Science, Rehovot, Israel; 225 Department of Physics, University of Wisconsin, Madison, WI USA; 226 Fakultät für Physik und Astronomie, Julius-Maximilians-Universität, Würzburg, Germany; 227 Fakultät für Mathematik und Naturwissenschaften, Fachgruppe Physik, Bergische Universität Wuppertal, Wuppertal, Germany; 228 Department of Physics, Yale University, New Haven, CT USA; 229 Yerevan Physics Institute, Yerevan, Armenia; 230 Centre de Calcul de l’Institut National de Physique Nucléaire et de Physique des Particules (IN2P3), Villeurbanne, France; 231 CERN, Geneva, Switzerland

## Abstract

This article documents the performance of the ATLAS muon identification and reconstruction using the LHC dataset recorded at $\sqrt{s} = 13$ TeV in 2015. Using a large sample of $J/\psi \rightarrow \mu \mu$ and $Z\rightarrow \mu \mu$ decays from 3.2 fb$^{-1}$ of *pp* collision data, measurements of the reconstruction efficiency, as well as of the momentum scale and resolution, are presented and compared to Monte Carlo simulations. The reconstruction efficiency is measured to be close to $99~\%$ over most of the covered phase space ($|\eta |<2.5$ and $5 < p_{\mathrm {T}} < 100$ GeV). The isolation efficiency varies between 93 and $100~\%$ depending on the selection applied and on the momentum of the muon. Both efficiencies are well reproduced in simulation. In the central region of the detector, the momentum resolution is measured to be $1.7~\%$ ($2.3~\%$) for muons from $J/\psi \rightarrow \mu \mu$ ($Z\rightarrow \mu \mu$) decays, and the momentum scale is known with an uncertainty of $0.05~\%$. In the region $|\eta |>2.2$, the $p_{\mathrm {T}}$ resolution for muons from $Z\rightarrow \mu \mu$ decays is $2.9~\%$ while the precision of the momentum scale for low-$p_{\mathrm {T}}$ muons from $J/\psi \rightarrow \mu \mu$ decays is about $0.2~\%$.

## Introduction

Muons are key to some of the most important physics results published by the ATLAS experiment [1] at the LHC. These results include the discovery of the Higgs boson [2] and the measurement of its properties [3–5], the precise measurement of Standard Model processes [6, 7], and searches for physics beyond the Standard Model [8–11].

The performance of the ATLAS muon reconstruction during the LHC run at $\sqrt{s}$ = 7–8 TeV has been documented in recent publications [12, 13]. During the 2013–2015 shutdown, the LHC was upgraded to increase the centre-of-mass energy from 8 to 13 TeV and the ATLAS detector was equipped with additional muon chambers and a new innermost Pixel layer, the Insertable B-Layer, providing measurements closer to the interaction point. Moreover, the muon reconstruction software was updated and improved.

After introducing the ATLAS muon reconstruction and identification algorithms, this article describes the performance of the muon reconstruction in the first dataset collected at $\sqrt{s}=13$ TeV. Measurements of the muon reconstruction and isolation efficiencies and of the momentum scale and resolution are presented. The comparison between data and Monte Carlo (MC) simulation and the determination of the corrections to the simulation used in physics analyses are also discussed. The results are based on the analysis of a large sample of ${J/\psi \rightarrow \mu \mu }$ and ${Z\rightarrow \mu \mu }$ decays reconstructed in 3.2 $\mathrm{fb}^{-1}$ of *pp* collisions recorded in 2015.

This article is structured as follows: Sect. 2 describes the ATLAS subdetectors that are most relevant to this work; Sects. 3 and 5 describe the muon reconstruction and identification in ATLAS, respectively; Sect. 4 describes the data samples used in the analysis; the reconstruction and isolation efficiencies are described in Sects. 6 and 7, respectively, while the momentum scale and resolution are described in Sect. 8. Finally, conclusions are given in Sect. 9.

## ATLAS detector

A detailed description of the ATLAS detector can be found in Ref. [1]. Information primarily from the inner detector (ID) and the muon spectrometer (MS), supplemented by information from the calorimeters, is used to identify and precisely reconstruct muons produced in *pp* collisions.

The ID consists of three subdetectors: the silicon pixels (Pixel) and the semiconductor tracker (SCT) with a pseudorapidity^1^ coverage up to $|\eta |$ = 2.5, and the transition radiation tracker (TRT) with a pseudorapidity coverage up to $|\eta |$ = 2.0. The ID measures the muon track close to the interaction point, providing accurate measurements of the track parameters inside an axial magnetic field of 2 T.

The MS is the outermost ATLAS subdetector. It is designed to detect muons in the pseudorapidity region up to $|\eta | = 2.7$, and to provide momentum measurements with a relative resolution better than 3 % over a wide $p_{\text {T}}$ range and up to 10 % at $p_{\text {T}} \approx 1$ TeV. The MS consists of one barrel ($|\eta | < 1.05$) and two endcap sections ($1.05 < |\eta | < 2.7$). A system of three large superconducting air-core toroidal magnets, each with eight coils, provides a magnetic field with a bending integral of about 2.5 Tm in the barrel and up to 6 Tm in the endcaps. Resistive plate chambers (RPC, three doublet layers for $|\eta | < 1.05$) and thin gap chambers (TGC, one triplet layer followed by two doublets for $1.0<|\eta | < 2.4$) provide triggering capability to the detector as well as ($\eta$, $\phi$) position measurements with typical spatial resolution of 5–10 mm. A precise momentum measurement for muons with pseudorapidity up to $|\eta |=2.7$ is provided by three layers of monitored drift tube chambers (MDT), with each chamber providing six to eight $\eta$ measurements along the muon trajectory. For $|\eta |>2$, the inner layer is instrumented with a quadruplet of cathode strip chambers (CSC) instead of MDTs. The single-hit resolution in the bending plane for the MDT and the CSC is about 80 and 60 $\upmu$m, respectively. The muon chambers are aligned with a precision between 30 and 60 $\upmu$m.

During the shutdown preceding the LHC Run 2, the MS was completed to its initial design [14] by adding the last missing chambers in the transition region between the barrel and the endcaps ($1.0<|\eta |<1.4$). Four RPC-equipped MDT chambers were also installed inside two elevator shafts to improve the acceptance in that region compared to Run 1. Some of the new MDT chambers are made of tubes with a smaller radius compared to the ones used in the rest of the detector, allowing the detector to cope with higher rates.

The material between the interaction point (IP) and the MS ranges approximately from 100 to 190 radiation lengths, depending on $\eta$, and consists mostly of calorimeters. The lead/liquid-argon electromagnetic calorimeter covers $|\eta |<3.2$. It is surrounded by hadronic calorimeters made of steel and scintillator tiles for $|\eta |< 1.7$, and copper or tungsten and liquid argon for $|\eta |> 1.7$.

## Muon reconstruction

Muon reconstruction is first performed independently in the ID and MS. The information from individual subdetectors is then combined to form the muon tracks that are used in physics analyses. In the ID, muons are reconstructed like any other charged particles as described in Refs. [15, 16]. This section focuses on the description of the muon reconstruction in the MS (Sect. 3.1) and on the combined muon reconstruction (Sect. 3.2).

### Muon reconstruction in the MS

Muon reconstruction in the MS starts with a search for hit patterns inside each muon chamber to form segments. In each MDT chamber and nearby trigger chamber, a Hough transform [17] is used to search for hits aligned on a trajectory in the bending plane of the detector. The MDT segments are reconstructed by performing a straight-line fit to the hits found in each layer. The RPC or TGC hits measure the coordinate orthogonal to the bending plane. Segments in the CSC detectors are built using a separate combinatorial search in the $\eta$ and $\phi$ detector planes. The search algorithm includes a loose requirement on the compatibility of the track with the luminous region.

Muon track candidates are then built by fitting together hits from segments in different layers. The algorithm used for this task performs a segment-seeded combinatorial search that starts by using as seeds the segments generated in the middle layers of the detector where more trigger hits are available. The search is then extended to use the segments from the outer and inner layers as seeds. The segments are selected using criteria based on hit multiplicity and fit quality and are matched using their relative positions and angles. At least two matching segments are required to build a track, except in the barrel–endcap transition region where a single high-quality segment with $\eta$ and $\phi$ information can be used to build a track.

The same segment can initially be used to build several track candidates. Later, an overlap removal algorithm selects the best assignment to a single track, or allows for the segment to be shared between two tracks. To ensure high efficiency for close-by muons, all tracks with segments in three different layers of the spectrometer are kept when they are identical in two out of three layers but share no hits in the outermost layer.

The hits associated with each track candidate are fitted using a global $\chi ^2$ fit. A track candidate is accepted if the $\chi ^2$ of the fit satisfies the selection criteria. Hits providing large contributions to the $\chi ^2$ are removed and the track fit is repeated. A hit recovery procedure is also performed looking for additional hits consistent with the candidate trajectory. The track candidate is refit if additional hits are found.

### Combined reconstruction

The combined ID–MS muon reconstruction is performed according to various algorithms based on the information provided by the ID, MS, and calorimeters. Four muon *types* are defined depending on which subdetectors are used in reconstruction:

- Combined (CB) muon: track reconstruction is performed independently in the ID and MS, and a combined track is formed with a global refit that uses the hits from both the ID and MS subdetectors. During the global fit procedure, MS hits may be added to or removed from the track to improve the fit quality. Most muons are reconstructed following an outside-in pattern recognition, in which the muons are first reconstructed in the MS and then extrapolated inward and matched to an ID track. An inside-out combined reconstruction, in which ID tracks are extrapolated outward and matched to MS tracks, is used as a complementary approach.
- Segment-tagged (ST) muons: a track in the ID is classified as a muon if, once extrapolated to the MS, it is associated with at least one local track segment in the MDT or CSC chambers. ST muons are used when muons cross only one layer of MS chambers, either because of their low $p_{\text {T}}$ or because they fall in regions with reduced MS acceptance.
- Calorimeter-tagged (CT) muons: a track in the ID is identified as a muon if it can be matched to an energy deposit in the calorimeter compatible with a minimum-ionizing particle. This type has the lowest purity of all the muon types but it recovers acceptance in the region where the ATLAS muon spectrometer is only partially instrumented to allow for cabling and services to the calorimeters and inner detector. The identification criteria for CT muons are optimised for that region ($|\eta |<0.1$) and a momentum range of $15< p_{\text {T}} < 100$ GeV.
- Extrapolated (ME) muons: the muon trajectory is reconstructed based only on the MS track and a loose requirement on compatibility with originating from the IP. The parameters of the muon track are defined at the interaction point, taking into account the estimated energy loss of the muon in the calorimeters. In general, the muon is required to traverse at least two layers of MS chambers to provide a track measurement, but three layers are required in the forward region. ME muons are mainly used to extend the acceptance for muon reconstruction into the region $2.5<|\eta |<2.7$, which is not covered by the ID.

Overlaps between different muon types are resolved before producing the collection of muons used in physics analyses. When two muon types share the same ID track, preference is given to CB muons, then to ST, and finally to CT muons. The overlap with ME muons in the muon system is resolved by analyzing the track hit content and selecting the track with better fit quality and larger number of hits.

The muon reconstruction used in this work evolved from the algorithms defined as *Chain 3* in Ref. [12]. These algorithms were improved in several ways. The use of a Hough transform to identify the hit patterns for seeding the segment-finding algorithm makes the reconstruction faster and more robust against misidentification of hadrons, thus providing better background rejection early in the pattern recognition process. The calculation of the energy loss in the calorimeter was also improved. An analytic parameterization of the average energy loss is derived from a detailed description of the detector geometry. The final estimate of the energy loss is obtained by combining the analytic parameterization with the energy measured in the calorimeter. This method yields a precision on the mean energy loss of about 30 MeV for 50 GeV muons.

## Data and Monte Carlo samples

The efficiency measurements presented in this article are obtained from the analysis of 3.2 $\mathrm{fb}^{-1}$ of *pp* collision data recorded at $\sqrt{s}=13$ TeV at the LHC in 2015 during the data-taking period with 25 ns spacing between bunch crossings. About $1.5\,\mathrm{M}$ ${Z\rightarrow \mu \mu }$ and $3.5\,\mathrm{M}$ ${J/\psi \rightarrow \mu \mu }$ events are reconstructed and used for the analysis. For the study of the momentum calibration, 2.7 $\mathrm{fb}^{-1}$ of data were used, rejecting the runs in which the longitudinal position of the beam spot was displaced by about 3 cm with respect to the centre of the detector.

Events are accepted only if the ID, the MS, and the calorimeters were operational and the solenoid and toroid magnet systems were both active. The online event selection was performed by a two-level trigger system derived from the one described in Ref. [18]. The $Z\rightarrow \mu \mu$ candidates are triggered by the presence of at least one muon candidate with a transverse momentum, $p_{\text {T}}$, of at least 20 GeV. For the reconstruction efficiency and momentum calibration studies, the muon firing the trigger is required to be isolated (see Sect. 7). The ${J/\psi \rightarrow \mu \mu }$ candidates used for the momentum calibration are triggered by a dedicated dimuon trigger that requires two opposite-charge muons, each with $p_{\text {T}} >4$ GeV, compatible with the same vertex, and with a dimuon invariant mass in the range 2.5–4.5 GeV. The ${J/\psi \rightarrow \mu \mu }$ sample used for the efficiency measurement is selected using a combination of single-muon triggers and triggers requiring one muon with transverse momentum of at least 4 GeV and an ID track such that the invariant mass of the muon+track pair, under a muon mass hypothesis, is compatible with the mass of the $J/\psi$.

Monte Carlo samples for the process $pp \rightarrow (Z/\gamma ^*) X \rightarrow \mu \mu X$ are generated using the POWHEG BOX [19] interfaced to PYTHIA8 [20] and the CT10 [21] parton distribution functions. The PHOTOS [22] package is used to simulate final-state photon radiation in *Z* boson decays. Samples of prompt ${J/\psi \rightarrow \mu \mu }$ decays are generated using PYTHIA8 complemented with PHOTOS to simulate the effects of final-state radiation. A requirement on the minimum transverse momentum of each muon ($p_{\text {T}} >4$ GeV) is applied at the generator level. The samples used for the simulation of the backgrounds to ${Z\rightarrow \mu \mu }$ include: $Z\rightarrow \tau \tau$, $W\rightarrow \mu \nu$, and $W\rightarrow \tau \nu$, generated with POWHEG BOX; *WW*, *ZZ*, and *WZ* generated with SHERPA [23]; $t\bar{t}$ samples generated with POWHEG BOX $+$ PYTHIA8; and $b\bar{b}$ and $c\bar{c}$ samples generated with PYTHIA8.

All the generated samples are passed through the simulation of the ATLAS detector based on GEANT4 [24, 25] and are reconstructed with the same programs used for the data. The ID and the MS are simulated with an ideal geometry assuming no misalignment.

The effect of multiple *pp* interactions per bunch crossing (“pile-up”) is modelled by overlaying simulated minimum-bias events onto the original hard-scattering event. Monte Carlo events are then reweighted so that the distribution of the average number of interactions per event agrees with the data.

## Muon identification

Muon identification is performed by applying quality requirements that suppress background, mainly from pion and kaon decays, while selecting prompt muons with high efficiency and/or guaranteeing a robust momentum measurement.

Muon candidates originating from in-flight decays of charged hadrons in the ID are often characterized by the presence of a distinctive “kink” topology in the reconstructed track. As a consequence, it is expected that the fit quality of the resulting combined track will be poor and that the momentum measured in the ID and MS may not be compatible. Several variables offering good discrimination between prompt muons and background muon candidates are studied in simulated $t\bar{t}$ events. Muons from *W* decays are categorized as *signal* muons while muon candidates from light-hadron decays are categorized as *background*. For CB tracks, the variables used in muon identification are:

- *q/p significance*, defined as the absolute value of the difference between the ratio of the charge and momentum of the muons measured in the ID and MS divided by the sum in quadrature of the corresponding uncertainties;
- $\rho ^{\prime }$, defined as the absolute value of the difference between the transverse momentum measurements in the ID and MS divided by the $p_{\text {T}}$ of the combined track;
- normalised $\chi ^2$ of the combined track fit.

To guarantee a robust momentum measurement, specific requirements on the number of hits in the ID and MS are used. For the ID, the quality cuts require at least one Pixel hit, at least five SCT hits, fewer than three Pixel or SCT holes, and that at least 10 % of the TRT hits originally assigned to the track are included in the final fit; the last requirement is only employed for $|\eta |$ between 0.1 and 1.9, in the region of full TRT acceptance. A hole is defined as an active sensor traversed by the track but containing no hits. A missing hit is considered a hole only when it falls between hits successfully assigned to a given track. If some inefficiency is expected for a given sensor, the requirements on the number of Pixel and SCT hits are reduced accordingly.

Four muon identification selections (*Medium*, *Loose*, *Tight*, and *High-p* $_\mathrm{T}$) are provided to address the specific needs of different physics analyses. *Loose*, *Medium*, and *Tight* are inclusive categories in that muons identified with tighter requirements are also included in the looser categories.

***Medium*** **muons** The *Medium* identification criteria provide the default selection for muons in ATLAS. This selection minimises the systematic uncertainties associated with muon reconstruction and calibration. Only CB and ME tracks are used. The former are required to have ${\ge }3$ hits in at least two MDT layers, except for tracks in the $|\eta |<0.1$ region, where tracks with at least one MDT layer but no more than one MDT hole layer are allowed. The latter are required to have at least three MDT/CSC layers, and are employed only in the $2.5<|\eta |<2.7$ region to extend the acceptance outside the ID geometrical coverage. A loose selection on the compatibility between ID and MS momentum measurements is applied to suppress the contamination due to hadrons misidentified as muons. Specifically, the *q/p significance* is required to be less than seven. In the pseudorapidity region $|\eta |<2.5$, about 0.5 % of the muons classified as *Medium* originate from the inside-out combined reconstruction strategy.

***Loose*** **muons** The *Loose* identification criteria are designed to maximise the reconstruction efficiency while providing good-quality muon tracks. They are specifically optimised for reconstructing Higgs boson candidates in the four-lepton final state [5]. All muon types are used. All CB and ME muons satisfying the *Medium* requirements are included in the *Loose* selection. CT and ST muons are restricted to the $|\eta |<0.1$ region. In the region $|\eta |<2.5$, about 97.5 % of the *Loose* muons are combined muons, approximately 1.5 % are CT and the remaining 1 % are reconstructed as ST muons.

***Tight*** **muons** *Tight* muons are selected to maximise the purity of muons at the cost of some efficiency. Only CB muons with hits in at least two stations of the MS and satisfying the *Medium* selection criteria are considered. The normalised $\chi ^2$ of the combined track fit is required to be ${<}8$ to remove pathological tracks. A two-dimensional cut in the $\rho ^{\prime }$ and *q/p significance* variables is performed as a function of the muon $p_{\text {T}}$ to ensure stronger background rejection for momenta below 20 GeV where the misidentification probability is higher.

***High-p*** $_\mathrm{T}$ **muons** The *High-p* $_\mathrm{T}$ selection aims to maximise the momentum resolution for tracks with transverse momentum above 100 GeV. The selection is optimised for searches for high-mass $Z'$ and $W'$ resonances [8, 9]. CB muons passing the *Medium* selection and having at least three hits in three MS stations are selected. Specific regions of the MS where the alignment is suboptimal are vetoed as a precaution. Requiring three MS stations, while reducing the reconstruction efficiency by about 20 %, improves the $p_{\text {T}}$ resolution of muons above 1.5 TeV by approximately 30 %.

The reconstruction efficiencies for *signal* and *background* obtained from $t\overline{t}$ simulation are reported in Table 1. The results are shown for the four identification selection criteria separating low ($4<p_{\text {T}} <20$ GeV) and high ($20<p_{\text {T}} <100$ GeV) transverse momentum muon candidates. No isolation requirement is applied in the selection shown in the table. When isolation requirements are applied, the misidentification rates are reduced by more than an order of magnitude. It should be noted that the higher misidentification rate observed for *Loose* with respect to *Medium* muons is mainly due to CT muons in the region $|\eta |<0.1$.

The misidentification probability estimated with the MC simulation is validated in data by measuring the probability that pions are reconstructed as muons. An unbiased sample of pions from $K_\mathrm{S}^0\rightarrow \pi ^+\pi ^-$ decays is collected with calorimeter-based (photon, electron, jet) triggers. Good agreement between data and simulation is observed independent of the $p_{\text {T}}$, $\eta$, and impact parameter of the track.

**Table 1:** Efficiency for prompt muons from *W* decays and hadrons decaying in-flight and misidentified as prompt muons computed using a $t\bar{t}$ MC sample. The results are shown for the four identification selection criteria separating low ($4<p_{\text {T}} <20$ GeV) and high ($20<p_{\text {T}} <100$ GeV) momentum muons for candidates with $|\eta |<2.5$. The statistical uncertainties are negligible

Selection	$4<p_{\text {T}} <20$ GeV		$20<p_{\text {T}} <100$ GeV	
	$\epsilon _{\mu }^{\mathrm {MC}}$ [%]	$\epsilon _{\mathrm {Hadrons}}^{\mathrm {MC}}$ [%]	$\epsilon _{\mu }^{\mathrm {MC}}$ [%]	$\epsilon _{\mathrm {Hadrons}}^{\mathrm {MC}}$ [%]
Loose	96.7	0.53	98.1	0.76
Medium	95.5	0.38	96.1	0.17
Tight	89.9	0.19	91.8	0.11
High-p$_\mathrm{T}$	78.1	0.26	80.4	0.13

## Reconstruction efficiency

As the muon reconstruction in the ID and MS detectors is performed independently, a precise determination of the muon reconstruction efficiency in the region $|\eta |<2.5$ is obtained with the tag-and-probe method, as described in the Sect. 6.1. A different methodology, described in Sect. 6.2, is used in the region $2.5<|\eta |<2.7$ where muons are reconstructed using only the MS detector.

### Efficiency measurement in the region $|\eta |<2.5$

The tag-and-probe method is employed to measure the efficiency of the muon identification selections within the acceptance of the ID ($|\eta |<2.5$). The method is based on the selection of an almost pure muon sample from ${J/\psi \rightarrow \mu \mu }$ or ${Z\rightarrow \mu \mu }$ events, requiring one leg of the decay (tag) to be identified as a *Medium* muon that fires the trigger and the second leg (probe) to be reconstructed by a system independent of the one being studied. A selection based on the event topology is used to reduce the background contamination.

Three kinds of probes are used to measure muon efficiencies. ID tracks and CT muons both allow a measurement of the efficiency in the MS, while MS tracks are used to determine the complementary efficiency of the muon reconstruction in the ID. Compared to ID tracks, CT muons offer a more powerful rejection of backgrounds, especially at low transverse momenta, and are therefore the preferred probe type for this part of the measurement. ID tracks are used as a cross-check and for measurements not directly accessible to CT muons. A direct measurement of the CT muon reconstruction efficiency is possible using MS tracks.

The efficiency measurement for *Medium*, *Tight*, and *High-p* $_\mathrm{T}$ muons consists of two stages. First, the efficiency $\epsilon \left( \text {X} | \text {CT}\right)$ (X $=$ *Medium*/*Tight*/*High-p* $_\mathrm{T}$) of reconstructing these muons assuming a reconstructed ID track is measured using a CT muon as probe. Then, this result is corrected by the efficiency $\epsilon \left( \text {ID} | \text {MS} \right)$ of the ID track reconstruction, measured using MS probes:

1 $$\begin{aligned} \epsilon \left( \text {X}\right)& =  \epsilon \left( \text {X} | \text {ID} \right) \cdot \epsilon \left( \text {ID}\right) = \epsilon \left( \text {X} | \text {CT} \right) \cdot \epsilon \left( \text {ID} | \text {MS}\right) \nonumber \\ & \quad ({\text {X}} = {\textit{Medium}}/{\textit{Tight}}/{\textit{High}}{\text{-}}{\textit{p}}_\mathrm{T}). \end{aligned}$$

A similar approach is used when using ID probe tracks for cross-checks.

This approach is valid if two assumptions are satisfied:

- the ID track reconstruction efficiency is independent from the muon spectrometer track reconstruction ($\epsilon \left( \text {ID}\right) = \epsilon \left( \text {ID} | \text {MS} \right)$).
- the use of a CT muon as a probe instead of an ID track does not affect the probability for *Medium*, *Tight*, or *High-p* $_\mathrm{T}$ reconstruction ($\epsilon \left( \text {X} | \text {ID} \right) = \epsilon \left( \text {X} | \text {CT} \right)$).

Both assumptions have been tested using generator-level information from simulation and small differences are taken into account in the systematic uncertainties.

The muons selected by the *Loose* identification requirements are decomposed into two samples: CT muons within $|\eta | < 0.1$ and all other muons. The CT muon efficiency is measured using MS probe tracks, while the efficiency of other muons is evaluated using CT probe muons in a fashion similar to the *Medium*, *Tight*, and *High-p* $_\mathrm{T}$ categories.

The level of agreement of the measured efficiency, $\epsilon ^{{\text {Data}}}\left( \text {X}\right)$, with the efficiency measured with the same method in simulation, $\epsilon ^{{\text {MC}}}\left( \text {X}\right)$, is expressed as the ratio of these two numbers, called the “efficiency scale factor” (SF):

2 $$\begin{aligned} \text {SF}=\frac{\epsilon ^{{\text {Data}}}\left( \text {X}\right) }{\epsilon ^{{\text {MC}}}\left( \text {X}\right) } \text {.} \end{aligned}$$

This quantity describes the deviation of the simulation from the real detector behaviour, and is of particular interest to physics analyses, where it is used to correct the simulation.

#### The tag-and-probe method with ${Z\rightarrow \mu \mu }$ events

Events are selected by requiring muon pairs with an invariant mass within $10~\mathrm{GeV}$ of the *Z* boson mass. The tag muon is required to satisfy the *Loose* isolation (see Sect. 7.2) and *Medium* muon identification selections and to have a transverse momentum of at least $24~\mathrm{GeV}$. Requirements on the significance of the transverse impact parameter $d_0$ ($|d_0|/\sigma (d_0) < 3.0$) and on the longitudinal impact parameter $|z_0|$ ($|z_0| < 10$ mm) of the tag muon are imposed. Finally, the tag muon is required to have triggered the readout of the event.

The probe muon is required to have a transverse momentum of at least $10~\mathrm{GeV}$ and to satisfy the *Loose* isolation criteria. While this is sufficient to ensure high purity in the case of MS probe tracks, further requirements are applied to both the ID track and CT muon probes. In the case of ID tracks, an isolation requirement is applied which is considerably stricter than the *Loose* selection in order to suppress backgrounds as much as possible. In addition, the invariant mass window is tightened to $5~\mathrm{GeV}$ around the *Z* boson mass, rather than the $10~\mathrm{GeV}$ used in the other cases. For CT muon probes, additional requirements on the compatibility of the associated calorimeter energy deposit with a muon signature are applied to further enhance the purity. The ID probe tracks and calorimeter-tagged probe muons must also have transverse and longitudinal impact parameters consistent with being produced in a primary *pp* interaction, as required for tag muons. A probe is considered successfully reconstructed if a reconstructed muon is found within a cone in the $\eta$–$\phi$ plane of size $\Delta R = 0.05$ around the probe track.

A small fraction (about 0.1 %) of the selected tag–probe pairs originates from sources other than $Z\rightarrow \mu \mu$ events. For a precise efficiency measurement, these backgrounds must be estimated and subtracted. Contributions from $Z\rightarrow \tau \tau$ and $t\bar{t}$ decays are estimated using simulation. Additionally, multijet events and $W\rightarrow \mu \nu$ decays in association with jet activity (*W*+jets) can yield tag–probe pairs through secondary muons from heavy- or light-hadron decays. As these backgrounds are approximately charge-symmetric, they are estimated from the data using same-charge (SC) tag–probe pairs. This leads to the following estimate of the opposite-charge (OC) background, $N^{\text {Bkg}}$, for each region of the kinematic phase-space:

3 $$\begin{aligned} N^{\text {Bkg}} = N_{\text {OC}}^{Z,t\bar{t}\text { MC}} + T \cdot \left( N_{\text {SC}}^{\text {Data}} - N_{\text {SC}}^{Z,t\bar{t}\text { MC}} \right) \end{aligned}$$

where $N_{\text {OC}}^{Z,t\bar{t}\text { MC}}$ is the contribution from $Z\rightarrow \tau \tau$ and $t\bar{t}$ decays, $N_{\text {SC}}^{\text {Data}}$ is the number of SC pairs measured in data and $N_{\text {SC}}^{Z,t\bar{t}\text { MC}}$ is the estimated contribution of the $Z\rightarrow \mu \mu$, $Z\rightarrow \tau \tau$, and $t\bar{t}$ processes to the SC sample. *T* is a global transfer factor that takes into account the charge asymmetry of the multijet and *W*+jets processes, estimated in data using a control sample of events obtained by inverting the probe isolation requirement. For MS (ID) tracks, a value of $T = 1.7$ (1.1) is obtained, while for calorimeter-tagged muon probes the transfer factor is $T = 1.2$. The systematic uncertainties in the transfer factor vary between 40 and 100 % and are included in the systematic error in the reconstruction efficiency described in Sect. 6.1.3.

The efficiency measured in the data is corrected for the background contributions described above by subtracting the predicted probe yields attributed to these sources from the number of observed probes,

4 $$\begin{aligned} \epsilon = \frac{N_{\text {R}}^{\text {Data}} - N_{\text {R}}^{\text {Bkg}}}{N_{\text {P}}^{\text {Data}} - N_{\text {P}}^{\text {Bkg}}}, \end{aligned}$$

where $N_{\text {P}}$ denotes the total number of probes and $N_{\text {R}}$ the number of successfully reconstructed probes. The resulting efficiency can then be compared directly to the result of the simulation.

#### The tag-and-probe method with ${J/\psi \rightarrow \mu \mu }$ events

The reconstruction efficiencies of the *Loose*, *Medium*, and *Tight* muon selections at low $p_{\text {T}}$ are measured from a sample of ${J/\psi \rightarrow \mu \mu }$ events selected using a combination of single-muon triggers and the dedicated “muon + track” trigger described in Sect. 4.

Tag–probe pairs are selected within the invariant mass window of 2.7–$3.5~\mathrm{GeV}$ and requiring a transverse momentum of at least $5~\mathrm{GeV}$ for each muon. The tag muon is required to satisfy the *Medium* muon identification selection and to have triggered the readout of the event. In order to avoid low-momentum curved tracks sharing the same trigger region, tag and probe muons are required to be $\Delta R > 0.2$ apart when extrapolated to the MS trigger surfaces. Finally, they are selected with $\Delta z_0 \equiv |z_0^{\mathrm {tag}} - z_0^{\mathrm {probe}}| < 5$ mm, to suppress background. A probe is considered successfully reconstructed if a selected muon is found within a $\Delta R = 0.05$ cone around the probe track.

The background contamination and the muon reconstruction efficiency are measured with a simultaneous maximum-likelihood fit of two statistically independent distributions of the invariant mass: events in which the probe is or is not successfully matched to the selected muon. The fits are performed in six $p_{\text {T}}$ and nine $\eta$ bins of the probe tracks. The signal is modelled with a Crystal Ball function [26] with a single set of parameters for the two independent samples. Separate first-order polynomial fits are used to describe the background shape for matched and unmatched probes.

#### Systematic uncertainties

**Fig. 1:**
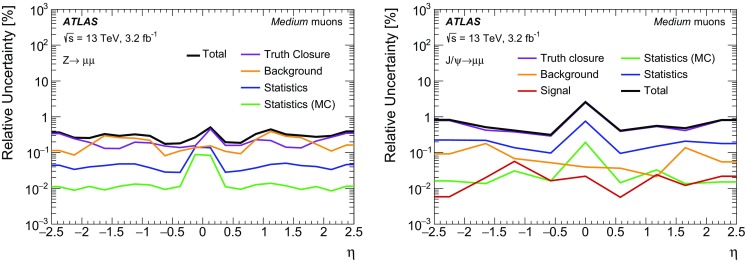
Total uncertainty in the efficiency scale factor for *Medium* muons as a function of $\eta$ as obtained from ${Z\rightarrow \mu \mu }$ data (*left*) for muons with $p_{\text {T}} >10$ GeV, and from ${J/\psi \rightarrow \mu \mu }$ data (*right*) for muons with $5<p_{\text {T}} <20$ GeV. The combined uncertainty is the sum in quadrature of the individual contributions

**Fig. 2:**
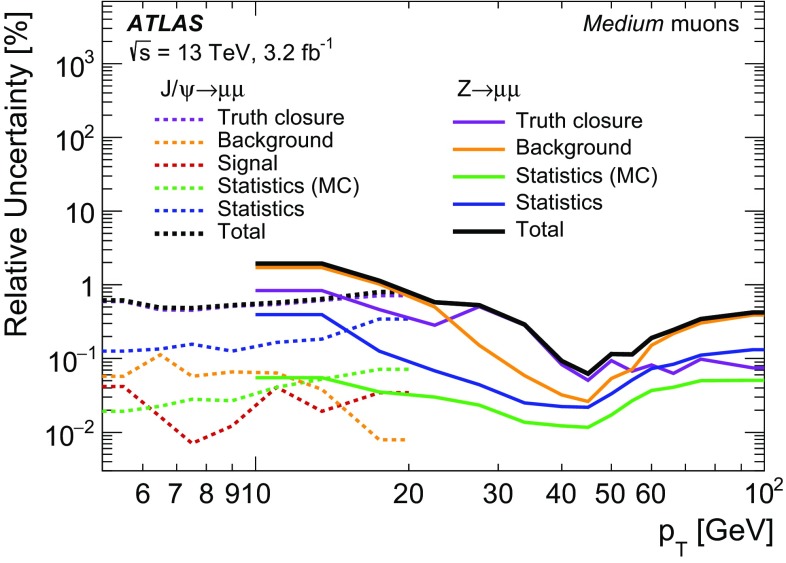
Total uncertainty in the efficiency scale factor for *Medium* muons as a function of $p_{\text {T}}$ as obtained from ${Z\rightarrow \mu \mu }$ (*solid lines*) and ${J/\psi \rightarrow \mu \mu }$ (*dashed lines*) decays. The combined uncertainty is the sum in quadrature of the individual contributions

The main contributions to the systematic uncertainty in the measurement of the efficiency SFs with ${Z\rightarrow \mu \mu }$ and ${J/\psi \rightarrow \mu \mu }$ events are shown in Figs. 1 and 2, as a function of $\eta$ and $p_{\text {T}}$, respectively.

The uncertainty in the background estimate is evaluated in the ${Z\rightarrow \mu \mu }$ analysis by taking the maximum variation of the transfer factor *T* when estimated with a simulation-based approach as described in Ref. [12] and when assuming the background to be charge-symmetric. This results in an uncertainty of the efficiency measurement below $0.1~\%$ over a large momentum range, but reaching ${\sim } 1~\%$ for low muon momenta where the contribution of the background is most significant. In the ${J/\psi \rightarrow \mu \mu }$ analysis, the background uncertainty is estimated by changing the function used in the fit to model the background, replacing the first-order polynomial with an exponential function. An uncertainty due to the signal modelling in the fit, labelled as “Signal” in Figs. 1 and 2, is also estimated using a convolution of exponential and Gaussian functions as an alternative model. Each uncertainty is about $0.1~\%$.

The cone size used for matching selected muons to probe tracks is optimised in terms of efficiency and purity of the matching. The systematic uncertainty deriving from this choice is evaluated by varying the cone size by ${\pm } 50~\%$. This yields an uncertainty below $0.1~\%$ in both analyses.

Possible biases in the tag-and-probe method, such as biases due to different kinematic distributions between reconstructed probes and generated muons or correlations between ID and MS efficiencies, are estimated in simulation by comparing the efficiency measured with the tag-and-probe method with the “true” efficiency given by the fraction of generator-level muons that are successfully reconstructed. This uncertainty is labelled as “Truth Closure” in Figs. 1 and 2. In the ${Z\rightarrow \mu \mu }$ analysis, agreement better than $0.1~\%$ is observed in the high momentum range. This uncertainty grows at low $p_{\text {T}}$, and differences up to $0.7~\%$ are found in the ${J/\psi \rightarrow \mu \mu }$ analysis. A larger effect of up to 1–2 $\%$ is measured in both analyses in the region $|\eta |<0.1$. In the extraction of the efficiency scale factors, the difference between the measured and the “true” efficiency cancels to first order. To take into account possible imperfections of the simulation, half of the observed difference is used as an additional systematic uncertainty in the SF.

No significant dependence of the measured SFs with $p_{\text {T}}$ is observed in the momentum range considered in the ${Z\rightarrow \mu \mu }$ analysis. An upper limit on the SF variation for large muon momenta is extracted from simulation, leading to an additional uncertainty of 2–3 % per TeV for muons with $p_{\text {T}} >200$ GeV. The efficiency scale factor is observed to be independent of the amount of pile-up.

#### Results

**Fig. 3:**
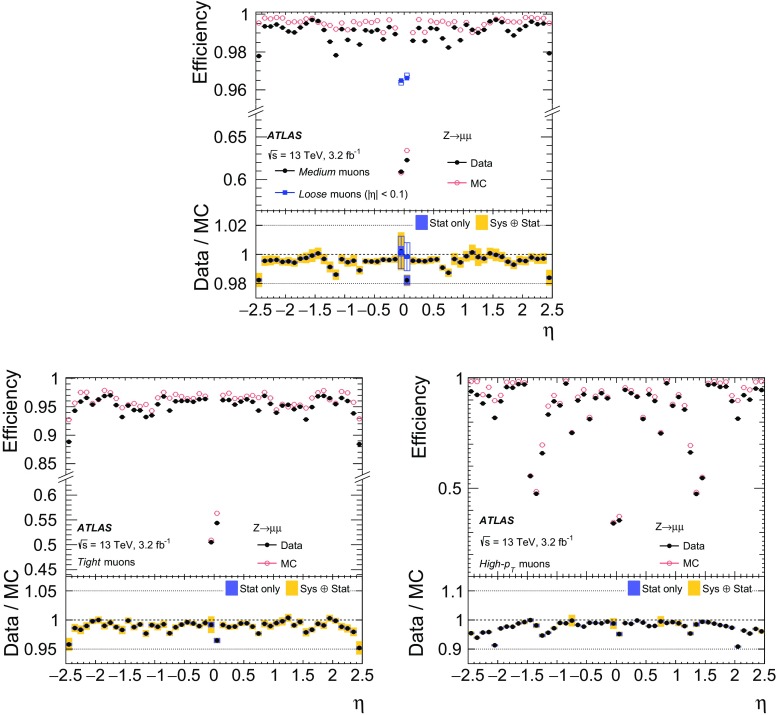
Muon reconstruction efficiency as a function of $\eta$ measured in ${Z\rightarrow \mu \mu }$ events for muons with $p_{\text {T}} >10$ GeV shown for *Medium* (*top*), *Tight* (*bottom left*), and *High-p* $_\mathrm{T}$ (*bottom right*) muon selections. In addition, the top plot also shows the efficiency of the *Loose* selection (*squares*) in the region $|\eta |<0.1$ where the *Loose* and *Medium* selections differ significantly. The *error bars* on the efficiencies indicate the statistical uncertainty. *Panels at the bottom* show the ratio of the measured to predicted efficiencies, with statistical and systematic uncertainties

**Fig. 4:**
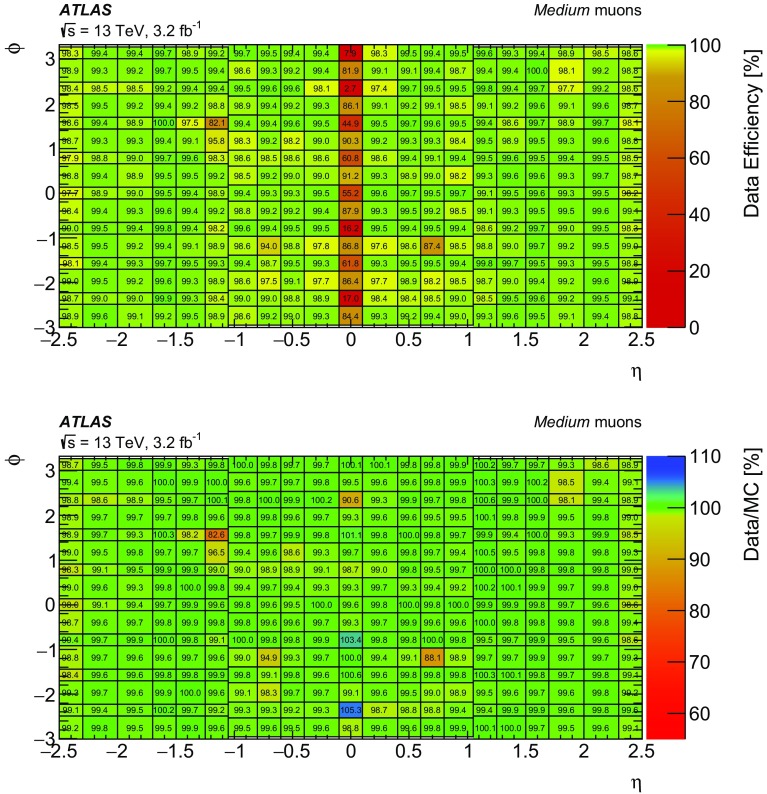
Reconstruction efficiency measured in data (*top*), and the data/MC efficiency scale factor (*bottom*) for *Medium* muons as a function of $\eta$ and $\phi$ for muons with $p_{\text {T}} >10$ GeV in ${Z\rightarrow \mu \mu }$ events. The *thin white bins* visible in the region $| \phi | \sim \pi$ are due to the different bin boundaries in $\phi$ in the endcap and barrel regions

**Fig. 5:**
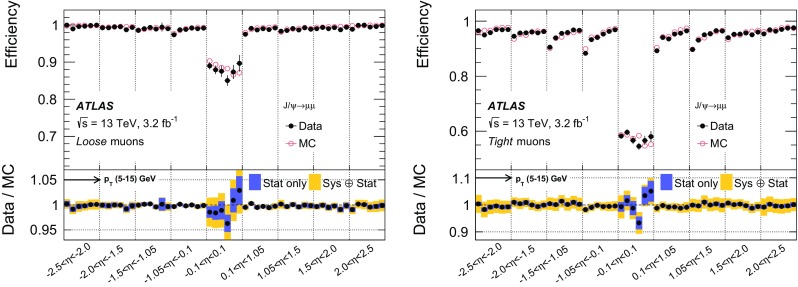
Muon reconstruction efficiency in different $\eta$ regions measured in ${J/\psi \rightarrow \mu \mu }$ events for *Loose* (*left*) and *Tight* (*right*) muon selections. Within each $\eta$ region, the efficiency is measured in six $p_{\text {T}}$ bins (5–6, 6–7, 7–8, 8–10, 10–12, and 12–15 GeV). The resulting values are plotted as distinct measurements in each $\eta$ bin with $p_{\text {T}}$ increasing from 5 to 15 GeV going from left to right. The *error bars* on the efficiencies indicate the statistical uncertainty. The *panel at the bottom* shows the ratio of the measured to predicted efficiencies, with statistical and systematic uncertainties

Figure 3 shows the muon reconstruction efficiency as a function of $\eta$ as measured from ${Z\rightarrow \mu \mu }$ events for the different muon selections. The efficiency as measured in data and the corresponding scale factors for the *Medium* selection are also shown in Fig. 4 as a function of $\eta$ and $\phi$. The efficiency at low $p_{\text {T}}$ is reported in Fig. 5 as measured from ${J/\psi \rightarrow \mu \mu }$ events as a function of $p_{\text {T}}$ in different $\eta$ regions.

The efficiencies of the *Loose* and *Medium* selections are very similar throughout the detector with the exception of the region $|\eta |<0.1$, where the *Loose* selection fills the MS acceptance gap using the calorimeter and segment-tagged muons contributions. The efficiency of these selections is observed to be in excess of $98~\%$, and between 90 and $98~\%$ for the *Tight* selection, with all efficiencies in very good agreement with those predicted by the simulation. An inefficiency due to a poorly aligned MDT chamber is clearly localised at $(\eta ,\phi ) \sim (-1.3, 1.6)$, and is the most significant feature of the comparison between collision data and simulation for these three categories. In addition, a $2~\%$-level local inefficiency is visible in the region $(\eta , \phi ) \sim (1.9, 2.5)$, traced to temporary failures in the SCT readout system. Further local inefficiencies in the barrel region around $\phi \sim -1.1$ are also linked to temporary faults during data taking. The efficiency of the *High-p* $_\mathrm{T}$ selection is significantly lower, as a consequence of the strict requirements on momentum resolution. Local disagreements between prediction and observation are more severe than in the case of the other muon selections. Apart from the poorly aligned MDT chamber, they are most prominent in the CSC region.

**Fig. 6:**
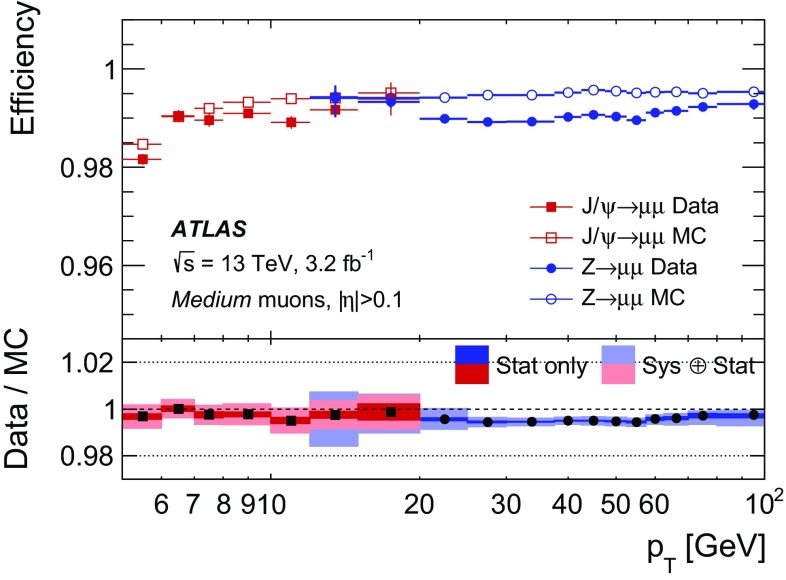
Reconstruction efficiency for the *Medium* muon selection as a function of the $p_{\text {T}}$ of the muon, in the region $0.1<|\eta |< 2.5$ as obtained with ${Z\rightarrow \mu \mu }$ and ${J/\psi \rightarrow \mu \mu }$ events. The *error bars* on the efficiencies indicate the statistical uncertainty. The *panel at the bottom* shows the ratio of the measured to predicted efficiencies, with statistical and systematic uncertainties

Figure 6 shows the reconstruction efficiencies for the *Medium* muon selection as a function of transverse momentum, including results from ${Z\rightarrow \mu \mu }$ and ${J/\psi \rightarrow \mu \mu }$, for muons with $0.1<|\eta |<2.5$. The efficiency is stable and slightly above 99 % for $p_{\text {T}} >6$ GeV. Values measured from ${J/\psi \rightarrow \mu \mu }$ and ${Z\rightarrow \mu \mu }$ events are in agreement in the overlap region between 10 and 20 GeV. The efficiency scale factors are also found to be compatible.

### Muon reconstruction efficiency for $|\eta |> 2.5$

As described in the previous sections, the reconstruction of combined muons is limited by the ID acceptance to the pseudorapidity region $|\eta |<2.5$. For $|\eta |>2.5$, the efficiency is recovered by using the ME muons included in the *Loose* and *Medium* muon selections. A measurement of the efficiency SF for muons in the region $2.5<|\eta |<2.7$ (high-$\eta$ region) is performed using the method described in Ref. [12]. The number of muons observed in ${Z\rightarrow \mu \mu }$ decays in the high-$\eta$ region is normalised to the number of muons observed in the region $2.2<|\eta |<2.5$. This ratio is calculated for both data and simulation, applying all known performance corrections to the region $|\eta |<2.5$. The SFs in the high-$\eta$ region are defined as the ratio of the aforementioned ratios and are provided in 4 $\eta$ and 16 $\phi$ bins. The values of the SFs measured using the 2015 dataset are close to 0.9 and are determined with a 3–5 % uncertainty.

## Isolation

Muons originating from the decay of heavy particles, such as *W*, *Z*, or Higgs bosons, are often produced isolated from other particles. Unlike muons from semileptonic decays, which are embedded in jets, these muons are well separated from other particles in the event. The measurement of the detector activity around a muon candidate, referred to as *muon isolation*, is therefore a powerful tool for background rejection in many physics analyses.

### Muon isolation variables

Two variables are defined to assess muon isolation: a track-based isolation variable and a calorimeter-based isolation variable.

The track-based isolation variable, $p_{\text {T}} ^{\mathrm {varcone30}}$, is defined as the scalar sum of the transverse momenta of the tracks with $p_{\text {T}} > 1$ GeV in a cone of size $\Delta R = {\mathrm {min}}\left( 10~\mathrm{GeV}/p_{\text {T}} ^{\mu }, 0.3\right)$ around the muon of transverse momentum $p_{\text {T}} ^{\mu }$, excluding the muon track itself. The cone size is chosen to be $p_{\text {T}}$-dependent to improve the performance for muons produced in the decay of particles with a large transverse momentum.

The calorimeter-based isolation variable, $E_{\text {T}} ^\mathrm {topocone20}$, is defined as the sum of the transverse energy of topological clusters [27] in a cone of size $\Delta R$ = 0.2 around the muon, after subtracting the contribution from the energy deposit of the muon itself and correcting for pile-up effects. Contributions from pile-up and the underlying event are estimated using the ambient energy-density technique [28] and are corrected on an event-by-event basis.

**Fig. 7:**
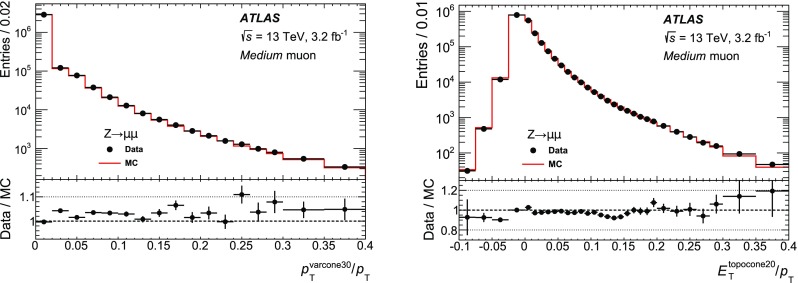
Distributions of the track-based (*left*) and the calorimeter-based (*right*) relative isolation variables measured in $Z\rightarrow \mu \mu$ events. Muons are selected by the *Medium* identification algorithm. The *dots* show the distribution for data while the histograms show the distribution from simulation. The *bottom panels* show the ratio of data to simulation with the corresponding statistical uncertainty. The pile-up reweighted simulated distribution is normalised to the number of events selected in data

The isolation selection criteria are determined using the *relative isolation variables*, which are defined as the ratio of the track- or calorimeter-based isolation variables to the transverse momentum of the muon. The distribution of the relative isolation variables in muons from ${Z\rightarrow \mu \mu }$ events is shown in the top panels of Fig. 7. Muons included in the plot satisfy the *Medium* identification criteria and are well separated from the other muon from the *Z* boson (${\Delta {}R}_{\mu \mu }>0.3$). The bottom panel shows the ratio of data to simulation.

### Muon isolation performance

Seven isolation selection criteria (*isolation working points*) are defined, each optimised for different physics analyses. Table 2 lists the seven isolation working points with the discriminating variables and the criteria used in their definition.

**Table 2:** Definition of the seven isolation working points. The discriminating variables are listed in the second column and the criteria used in the definition are reported in the third column

Isolation WP	Discriminating variable(s)	Definition
*LooseTrackOnly*	$p_{\text {T}} ^{\mathrm {varcone30}}/p_{\text {T}} ^{\mu }$	99 % efficiency constant in $\eta$ and $p_{\text {T}}$
*Loose*	$p_{\text {T}} ^{\mathrm {varcone30}}/p_{\text {T}} ^{\mu }$, $E_{\text {T}} ^{\mathrm {topocone20}}/p_{\text {T}} ^{\mu }$	99 % efficiency constant in $\eta$ and $p_{\text {T}}$
*Tight*	$p_{\text {T}} ^{\mathrm {varcone30}}/p_{\text {T}} ^{\mu }$, $E_{\text {T}} ^{\mathrm {topocone20}}/p_{\text {T}} ^{\mu }$	96 % efficiency constant in $\eta$ and $p_{\text {T}}$
*Gradient*	$p_{\text {T}} ^{\mathrm {varcone30}}/p_{\text {T}} ^{\mu }$, $E_{\text {T}} ^{\mathrm {topocone20}}/p_{\text {T}} ^{\mu }$	${\ge } 90 (99)~\%$ efficiency at 25 (60) GeV
*GradientLoose*	$p_{\text {T}} ^{\mathrm {varcone30}}/p_{\text {T}} ^{\mu }$, $E_{\text {T}} ^{\mathrm {topocone20}}/p_{\text {T}} ^{\mu }$	${\ge } 95 (99)~\%$ efficiency at 25 (60) GeV
*FixedCutTightTrackOnly*	$p_{\text {T}} ^{\mathrm {varcone30}}/p_{\text {T}} ^{\mu }$	$p_{\text {T}} ^{\mathrm {varcone30}}/p_{\text {T}} ^{\mu } < 0.06$
*FixedCutLoose*	$p_{\text {T}} ^{\mathrm {varcone30}}/p_{\text {T}} ^{\mu }$, $E_{\text {T}} ^{\mathrm {topocone20}}/p_{\text {T}} ^{\mu }$	$p_{\text {T}} ^{\mathrm {varcone30}}/p_{\text {T}} ^{\mu }<0.15$, $E_{\text {T}} ^{\mathrm {topocone20}}/p_{\text {T}} ^{\mu }<0.30$

**Fig. 8:**
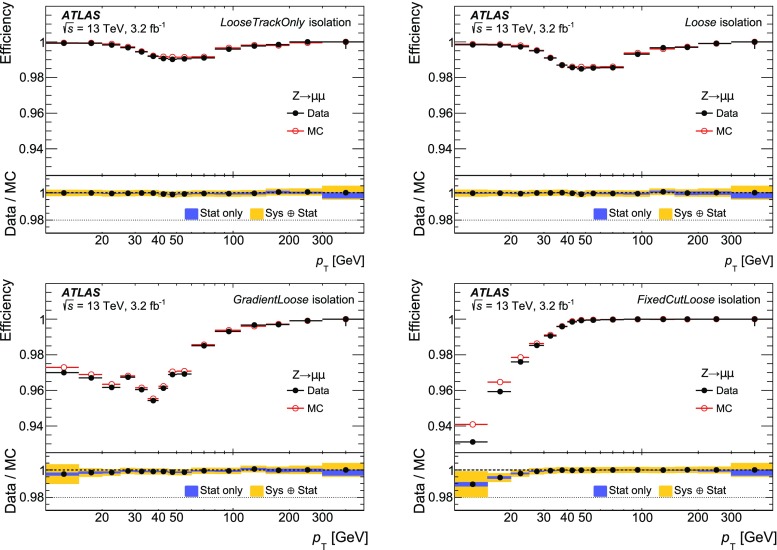
Isolation efficiency for the *LooseTrackOnly* (*top left*), *Loose* (*top right*), *GradientLoose* (*bottom left*), and *FixedCutLoose* (*bottom right*) muon isolation working points. The efficiency is shown as a function of the muon transverse momentum $p_{\text {T}}$ and is measured in $Z\rightarrow \mu \mu$ events. The *full* (*empty*) *markers* indicate the efficiency measured in data (MC) samples. The errors shown on the efficiency are statistical only. The *bottom panel* shows the ratio of the efficiency measured in data and simulation, as well as the statistical uncertainties and combination of statistical and systematic uncertainties

The efficiencies for the seven isolation working points are measured in data and simulation in ${Z\rightarrow \mu \mu }$ decays using the tag-and-probe method described in Sect. 6. To avoid probe muons in the vicinity of a jet, the angular separation ${\Delta {}R}$ between the probe muon and the closest jet, reconstructed using an anti-$k_t$ algorithm [29] with radius parameter 0.4 and with a transverse momentum greater than 20 GeV, is required to be greater than 0.4. In addition, the two muons originating from the *Z* boson decay are required to be separated by ${\Delta {}R}_{\mu \mu }>0.3$. Figure 8 shows the isolation efficiency measured for *Medium* muons in data and simulation as a function of the muon $p_{\text {T}}$ for the *LooseTrackOnly*, *Loose*, *GradientLoose*, and *FixedCutLoose* working points, with the respective data/MC ratios included in the bottom panel. The systematic uncertainties in the SFs are estimated by varying the background contributions within their uncertainties and by varying some of the selection criteria, such as the invariant mass selection window, the isolation of the tag muon, the minimum quality of the probe muon, the opening angle between the two muons, and the $\Delta R$ between the probe muon and the closest jet. In Fig. 8, the largest systematic uncertainty contribution over the entire $p_{\text {T}}$ region arises from having neglected the $\eta$ dependence of the SFs, which are usually provided as a function of $\eta$ and $p_{\text {T}}$. In the low-$p_{\text {T}}$ region, other important contributions are due to the background estimation and the mass window variation, while the high-$p_{\text {T}}$ region is dominated by statistical uncertainties in data and simulation. The total uncertainty is at the per mille level over a wide range of $p_{\text {T}}$ and reaches the percent level in the high-$p_{\text {T}}$ region. The suppression factor for muons from light mesons or *b*/*c* semileptonic decays is estimated from simulation and depends on the isolation working point, ranging from a minimum of 15 for *LooseTrackOnly* to a maximum of 40 for *Gradient*.

## Momentum scale and resolution

The muon momentum scale and resolution are studied using ${J/\psi \rightarrow \mu \mu }$ and ${Z\rightarrow \mu \mu }$ decays. Although the simulation contains an accurate description of the ATLAS detector, the level of detail is not enough to describe the muon momentum scale to the per mille level and the muon momentum resolution to the percent level. To obtain such a level of agreement between data and simulation, a set of corrections is applied to the simulated muon momentum. The methodology used to extract these corrections is described in Sect. 8.1. In Sect. 8.2, measurements of the muon momentum scale and resolution in data and simulation are presented for various detector regions and for a wide range of $p_{\text {T}}$. To improve the precision of the procedure, the $p_{\text {T}}$ and $\eta$ distributions of the *Z* and $J/\psi$ resonances in simulation are reweighted to the distributions observed in data.

### Muon momentum calibration procedure

In the following, the “muon momentum calibration” is defined as the procedure used to identify the corrections to the simulated muon transverse momenta reconstructed in the ID and MS subdetectors to precisely describe the measurement of the same quantities in data. Only CB muons are used to extract the calibration parameters. The transverse momentum of the ID and MS components of a CB track, referred to as $p_{\text {T}} ^{\text {ID}}$ and $p_{\text {T}} ^{\text {MS}}$, respectively, are used. The ID (MS) tracks are reconstructed using the hits from the ID (MS) detector and are extrapolated to the interaction point. In the case of MS tracks, the fit corrects for the energy loss in the calorimeters as described earlier.

The corrected transverse momentum, $p_{\text {T}} ^\mathrm{Cor,Det}$ ($\mathrm{Det} = \mathrm{ID, MS}$), is described by the following equation:

5 $$\begin{aligned} p_{\text {T}} ^\mathrm{Cor,Det}= & {} \frac{p_{\text {T}} ^\mathrm{MC,Det} + \sum \limits ^{1}_{n=0} s^\mathrm{Det}_{n}(\eta ,\phi )\left( p_{\text {T}} ^\mathrm{MC,Det}\right) ^{n}}{1+\sum \limits ^{2}_{m=0} \Delta r^\mathrm{Det}_{m}(\eta ,\phi )\left( p_{\text {T}} ^\mathrm{MC,Det}\right) ^{m-1}g_{m}}, \end{aligned}$$

where $p_{\text {T}} ^\mathrm{MC, Det}$ is the uncorrected transverse momentum in simulation, $g_{m}$ are normally distributed random variables with zero mean and unit width, and the terms $\Delta r_{m}^\mathrm{Det}(\eta ,\phi )$ and $s_n^\mathrm{Det}(\eta , \phi )$ describe the momentum resolution smearing and the scale corrections applied in a specific ($\eta$, $\phi$) detector region, respectively.

The corrections described in Eq. (5) are defined in $\eta$–$\phi$ detector regions that are homogeneous in terms of detector technology and performance. Both the ID and the MS are divided into 18 pseudorapidity regions. In addition, the MS is divided into two $\phi$ bins separating the two types of $\phi$ sectors: those that include the magnet coils (*small sectors*) and those between two coils (*large sectors*). The small and large MS sectors employ independent alignment techniques and cover detector areas with different material distribution. Therefore, relevant scale and resolution differences exist.

The numerator of Eq. (5) describes the momentum scales. The $s_1^\mathrm{Det}$ term corrects for inaccuracy in the description of the magnetic field integral and the dimension of the detector in the direction perpendicular to the magnetic field. The $s^\mathrm{MS}_0(\eta , \phi )$ term models the effect on the MS momentum from the inaccuracy in the simulation of the energy loss in the calorimeter and other materials between the interaction point and the MS. As the energy loss between the interaction point and the ID is negligible, $s^\mathrm{ID}_0(\eta )$ is set to zero.

The denominator of Eq. (5) describes the momentum smearing that broadens the relative $p_{\text {T}}$ resolution in simulation, $\sigma (p_{\text {T}})/ p_{\text {T}}$, to properly describe the data. The corrections to the resolution assume that the relative $p_{\text {T}}$ resolution can be parameterized as follows:

6 $$\begin{aligned} \frac{ \sigma (p_{\text {T}})}{p_{\text {T}}} = r_0/p_{\text {T}} \oplus r_1 \oplus r_2\cdot p_{\text {T}} \text {,} \end{aligned}$$

with $\oplus$ denoting a sum in quadrature. In Eq. (6), the first term accounts mainly for fluctuations of the energy loss in the traversed material, the second term accounts mainly for multiple scattering, local magnetic field inhomogeneities and local radial displacements of the hits, and the third term mainly describes intrinsic resolution effects caused by the spatial resolution of the hit measurements and by residual misalignment of the muon spectrometer. The energy loss term is negligible in both the ID and MS measurements, and therefore $\Delta r^\mathrm{ID}_{0}$ and $\Delta r^\mathrm{MS}_{0}$ are set to zero.

The corrected momentum of the combined muons, $p_{\text {T}} ^\mathrm{Cor,CB}$, is obtained by combining the ID and MS corrected momenta using a weighted average:

7 $$\begin{aligned} p_{\text {T}} ^\mathrm{Cor,CB} = f\cdot p_{\text {T}} ^\mathrm{Cor,ID} + (1 - f)\cdot p_{\text {T}} ^\mathrm{Cor, MS}\text {,} \end{aligned}$$

with the weight *f* derived from the following linear equation

8 $$\begin{aligned} p_{\text {T}} ^\mathrm{MC,CB} = f\cdot p_{\text {T}} ^\mathrm{MC,ID} + (1 - f)\cdot p_{\text {T}} ^\mathrm{MC, MS} \end{aligned}$$

which assumes that the relative contribution of the two subdetectors to the combined track remains unchanged before and after momentum corrections.

#### Determination of the $p_{\text {T}}$ calibration constants

The MS and ID correction parameters contained in Eq. (5) are extracted from data using a binned maximum-likelihood fit with templates derived from simulation which compares the invariant mass distributions for ${J/\psi \rightarrow \mu \mu }$ and ${Z\rightarrow \mu \mu }$ candidates in data and simulation. The exceptions are $\Delta r^\mathrm{ID}_{0}$, $\Delta r^\mathrm{MS}_{0}$, and $s^\mathrm{ID}_0$, which are set to zero, and $\Delta r^\mathrm{MS}_{2}$, which is determined from alignment studies using special runs with the toroidal magnetic field off.

The ${J/\psi \rightarrow \mu \mu }$ and ${Z\rightarrow \mu \mu }$ candidates are selected by requiring two oppositely charged CB muons satisfying the *Medium* identification criteria. Both muons must have impact parameters compatible with tracks produced by the primary interaction and pseudorapidity within the acceptance of both the ID and MS detectors $(|\eta |<2.5).$ Both muons from ${J/\psi \rightarrow \mu \mu }$ (${Z\rightarrow \mu \mu }$) candidate decays are required to have momenta in the range 5–20 (22–300) GeV and to form an invariant mass in the range 2.65–3.6 (76–106) GeV. Muons from *Z* boson decays need to be isolated, while no isolation criterion is imposed on muons from $J/\psi$ decays.

The extraction of the correction parameters is performed in $\eta$–$\phi$ regions of fit (ROFs) defined separately for the ID and the MS. Events are assigned to a specific ROF if at least one muon falls in the corresponding $\eta$–$\phi$ region.

The ID corrections are extracted using the distributions of the ID dimuon invariant mass, $m_{\mu \mu }^{\text {ID}}$. To enhance the sensitivity to $p_{\text {T}}$-dependent correction effects, the $m_{\mu \mu }^{\text {ID}}$ is classified according to the $p_{\text {T}}$ of the muons. For ${J/\psi \rightarrow \mu \mu }$ (${Z\rightarrow \mu \mu }$) decays, the fit is performed in two exclusive categories defined requiring the candidates to have $p_{\text {T}} ^{\text {ID}}$ of the sub-leading (leading) muon greater than 5 or 9 (22 or 47) GeV, respectively.

Similarly, the MS corrections are extracted using the distributions of the MS-reconstructed dimuon invariant mass, $m_{\mu \mu }^{\text {MS}}$. Since there are more parameters and more ROFs in the MS version of Eq. (5), an additional variable is added to the MS fit. This is defined by the following equation

9 $$\begin{aligned} \rho = \frac{p_{\text {T}} ^{\text {MS}} - p_{\text {T}} ^{\text {Cor,ID}}}{p_{\text {T}} ^{\text {Cor,ID}}}, \end{aligned}$$

which represents the $p_{\text {T}}$ imbalance between the measurement in the ID and in the MS. In Eq. (9), the momentum of the ID, $p_\mathrm{T}^{\mathrm {Cor,ID}}$, contains the appropriate $p_{\text {T}}$ corrections. The variable $\rho$ is used only in ${Z\rightarrow \mu \mu }$ candidate events and is binned according to $p_{\text {T}} ^{\text {MS}}$ of the muon with lower bin boundaries of $p_{\text {T}} ^{\text {MS}}={22, 35, 47, 60, 90}$ GeV.

Templates for the $m_{\mu \mu }^{\text {ID}}$, $m_{\mu \mu }^{\text {MS}}$, and $\rho$ are built using ${J/\psi \rightarrow \mu \mu }$ and ${Z\rightarrow \mu \mu }$ simulated signal samples. In the ${Z\rightarrow \mu \mu }$ sample, the small background component (approximately 0.1 %) is extracted from simulation and added to the templates. A much larger (about 15 %) non-resonant background from decays of light and heavy hadrons and from continuum Drell–Yan production is present in the ${J/\psi \rightarrow \mu \mu }$ sample. As this background is not easy to simulate, a data-driven approach is used. The dimuon invariant mass distribution in data is fitted in each ROF using a Crystal Ball function added to an exponential background distribution in the ID and MS fits. The background model and its normalisation are then used in the template fit.

The results are shown in Tables 3 and 4, averaged over three $\eta$ regions. The quoted errors include systematic uncertainties evaluated by varying several parameters of the template fit. The main contributions to the final systematic uncertainty are:

- Mass window width for the ${Z\rightarrow \mu \mu }$ candidate selection. Non-Gaussian smearing effects are accounted for by varying the $m_{\mu \mu }$ selection by $\pm 5$ GeV.
- Background parameterization for the $J/\psi$ fit as well as increased muon $p_{\text {T}}$ cut (from 5 to 7 GeV) to reduce the weight of the contribution of low-$p_{\text {T}}$ muons.
- Scale parameter for the ID corrections obtained by fitting separately the ${J/\psi \rightarrow \mu \mu }$ and ${Z\rightarrow \mu \mu }$ samples, to include possible non-linear scale effects.
- As $\Delta r_2^{\mathrm {MS}}$ is sensitive to the alignment of the MS chambers, its systematic uncertainty is determined from alignment studies performed on special runs where the toroidal magnetic field was turned off.

**Table 3:** Summary of ID muon momentum resolution and scale corrections used in Eq. (5), averaged over three main detector regions. The corrections are derived in 18 pseudorapidity regions, as described in Sect. 8, and averaged, assigning a weight to each region proportional to its $\eta$ width. The uncertainties represent the sum in quadrature of the statistical and systematic uncertainties

Region	$\Delta r_1^{\text {ID}}(\times 10^{-3})$	$\Delta r_2^{\text {ID}}$ [TeV$^{-1}$]	$s_1^{\text {ID}}(\times 10^{-3})$
$|\eta |<1.05$	$4.1\phantom {}^{+0.6}_{-0.9}$	$0.17 \phantom {}^{+0.04}_{-0.03}$	$-0.6\phantom {}^{+0.1}_{-0.2}$
$1.05\le |\eta |<2.0$	$5.5\phantom {}^{+2.5}_{-0.8}$	$0.34\phantom {}^{+0.07}_{-0.09}$	$-0.5 \phantom {}^{+0.2}_{-0.5}$
$|\eta |\ge 2.0$	$9 \phantom {}^{+9}_{-2}$	$0.05 \pm {0.01}$	$1.0 \phantom {}^{+3.5}_{-1.6}$

**Table 4:** Summary of MS momentum resolution and scale corrections for small and large MS sectors, averaged over three main detector regions. The corrections for large and small MS sectors are derived in 18 pseudorapidity regions, as described in Sect. 8, and averaged assigning a weight to each region proportional to its $\eta$ width. The energy loss term $\Delta r_0^{\text {MS}}$ is negligible and therefore fixed to zero in the fit for all $\eta$. The uncertainties represent the sum in quadrature of the statistical and systematic uncertainties

Region	$\Delta r_1^{\text {MS}} (\times 10^{-3})$	$\Delta r_2^{\text {MS}}$ [TeV$^{-1}$]	$s_0^{\text {MS}}$ [MeV]	$s_1^{\text {MS}} (\times 10^{-3})$
$|\eta |<1.05$ (small)	$17 \pm {1}$	$0.080 \pm {0.006}$	$-23 \pm {5}$	$-0.9 \pm {0.3}$
$|\eta |<1.05$ (large)	$15 \pm {1}$	$0.162 \pm {0.007}$	$-26 \phantom {}^{+8}_{-5}$	$1.8\phantom {}^{+0.4}_{-0.3}$
$1.05\le |\eta |<2.0$ (small)	$25 \phantom {}^{+3}_{-1}$	$0.20 \pm {0.03}$	$-13 \pm {6}$	$-1.4\pm {0.4}$
$1.05\le |\eta |<2.0$ (large)	$23 \phantom {}^{+3}_{-1}$	$0.160 \pm {0.015}$	$-15 \pm {10}$	$-1.1 \phantom {}^{+0.5}_{-0.6}$
$|\eta |\ge 2.0$ (small)	$17 \phantom {}^{+3}_{-1}$	$0.08 \pm {0.01}$	$-6 \phantom {}^{+6}_{-7}$	$0.7 \phantom {}^{+0.4}_{-0.3}$
$|\eta |\ge 2.0$ (large)	$15 \phantom {}^{+4}_{-3}$	$0.112 \pm {0.010}$	$-3 \phantom {}^{+13}_{-10}$	$0.3 \phantom {}^{+0.6}_{-0.7}$

### Dimuon mass scale and resolution after applying momentum corrections

The samples of ${J/\psi \rightarrow \mu \mu }$ and ${Z\rightarrow \mu \mu }$ decays are used to study the muon momentum scales and resolution in data and simulation and to validate the momentum corrections obtained with the template fit method described in the previous section.

**Fig. 9:**
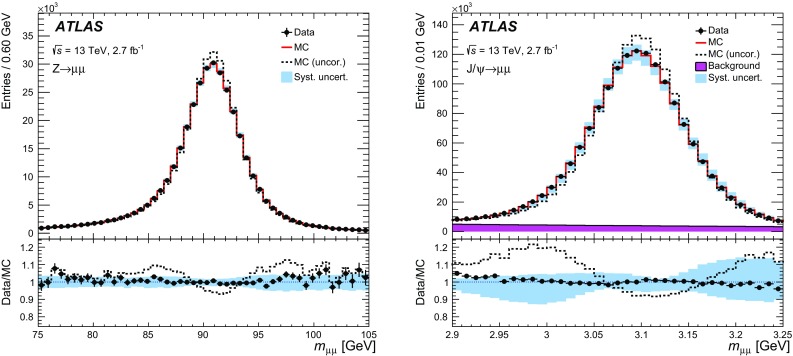
Dimuon invariant mass distribution of $Z\rightarrow \mu \mu$ (*left*) and $J/\psi \rightarrow \mu \mu$ (*right*) candidate events reconstructed with CB muons. The *upper panels* show the invariant mass distribution for data and for the signal simulation plus the background estimate. The points show the data. The *continuous line* corresponds to the simulation with the MC momentum corrections applied while the *dashed lines* show the simulation when no correction is applied. Background estimates are added to the signal simulation. The band represents the effect of the systematic uncertainties on the MC momentum corrections. The *lower panels* show the data to MC ratios. In the *Z* sample, the MC background samples are added to the signal sample according to their expected cross sections. In the $J/\psi$ sample, the background is estimated from a fit to the data as described in the text. The sum of background and signal MC distributions is normalised to the data

The invariant mass distributions for the ${J/\psi \rightarrow \mu \mu }$ and ${Z\rightarrow \mu \mu }$ candidates are shown in Fig. 9 and compared with uncorrected and corrected simulation. In the uncorrected simulation, it is noticeable that the signal distributions are narrower and slightly shifted with respect to data. After correction, the lineshapes of the two resonances in simulation agree with the data within the systematic uncertainties, demonstrating the overall effectiveness of the $p_{\text {T}}$ calibration.

A better demonstration of the effectiveness of the momentum calibration is obtained by comparing, in data and simulation, the measurement of the position $m_{\mu \mu }$ and resolution $\sigma _{\mu \mu }$ of the dimuon mass peaks, extracted in bins of $\eta$ and $p_{\text {T}}$ from fits to the ${J/\psi \rightarrow \mu \mu }$ and ${Z\rightarrow \mu \mu }$ invariant mass distributions.

When the two muons have similar momentum resolution and angular effects are neglected, the relative mass resolution, $\sigma _{\mu \mu }/m_{\mu \mu }$, is directly proportional to the relative muon momentum resolution, $\sigma _{p_{\mu }}/p_{\mu }$:

10 $$\begin{aligned} \frac{\sigma _{\mu \mu }}{m_{\mu \mu }} \, = \, \frac{1}{\sqrt{2}} \frac{\sigma _{p_{\mu }}}{p_{\mu }}. \end{aligned}$$

Similarly, the total muon momentum scale, defined as $s = \langle (p^\mathrm{meas} - p^\mathrm{true})/ p^\mathrm{true} \rangle$, is directly related to the dimuon mass scale, defined as $s_{\mu \mu } = \langle (m_{\mu \mu }^\mathrm{meas} - m_{\mu \mu }^\mathrm{true})/ m_{\mu \mu }^\mathrm{true} \rangle$:

11 $$\begin{aligned} s_{\mu \mu } = \sqrt{s_{\mu _1}s_{\mu _2}}, \end{aligned}$$

where $s_{\mu _1}$ and $s_{\mu _2}$ are the momentum scales of the two muons.

The dimuon mass resolution is obtained by fitting the width of the invariant mass peaks. In ${J/\psi \rightarrow \mu \mu }$ decays, the intrinsic width of the resonance is negligible with respect to the experimental resolution. The bulk of the peak is modelled by a Crystal Ball function; a Gaussian distribution centred on the Crystal Ball function is added to the signal description to model the tails of the distribution. The non-resonant background is described by an exponential function. In ${Z\rightarrow \mu \mu }$ decays, the fits use a convolution of the true lineshape (modelled by a Breit–Wigner function) with an experimental resolution function (a combination of a Crystal Ball and a Gaussian function). Similarly to the $J/\psi$, the non-resonant background is described by an exponential function. The peak position and width of the Crystal Ball function are used as estimators for the $m_{\mu \mu }$ and $\sigma (m_{\mu \mu })$ variables in the various $\eta$ and $p_{\text {T}}$ bins.

**Fig. 10:**
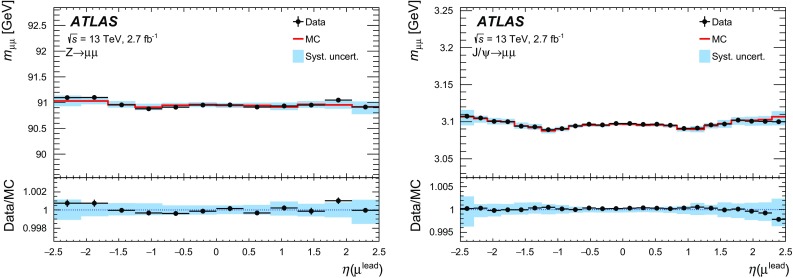
Fitted mean mass of the dimuon system for CB muons for ${Z\rightarrow \mu \mu }$ (*left*) and ${J/\psi \rightarrow \mu \mu }$ (*right*) events for data and corrected simulation as a function of the pseudorapidity of the highest-$p_{\text {T}}$ muon. The *upper panels* show the fitted mean mass value for data and corrected simulation. The small variations of the invariant mass estimator as a function of pseudorapidity are due to imperfect energy loss corrections and magnetic field description in the muon reconstruction. Both effects are well reproduced in the simulation. The *lower panels* show the data/MC ratio. The *error bars* represent the statistical uncertainty; the *shaded bands* represent the systematic uncertainty in the correction and the systematic uncertainty in the extraction method added in quadrature

Figure 10 shows the position of the peak of the invariant mass distribution, $m_{\mu \mu }$, obtained from the fits to the *Z* boson and $J/\psi$ samples as a function of the pseudorapidity of the highest-$p_{\text {T}}$ muon for CB pairs. The distributions are shown for data as well as corrected simulation, with the ratio of the two in the lower panel. The simulation is in very good agreement with the data. Minor deviations are contained within the scale systematic uncertainties of $0.05~\%$ in the barrel region, increasing with $|\eta |$ to $0.1~\% (0.3~\%)$ in the region $|\eta |\sim 2.5$ for ${Z\rightarrow \mu \mu }$ (${J/\psi \rightarrow \mu \mu }$) decays. The systematic uncertainties shown in the plots include the effects of the uncertainties in the calibration constants described in Sect. 8.1 and the changes in the fit parameterization. The observed level of agreement demonstrates that the $p_{\text {T}}$ calibration for combined muon tracks described above provides a very accurate description of the momentum scale in all $\eta$ regions, over a wide $p_{\text {T}}$ range. Similar levels of data/MC agreement are observed for the ID and MS components of the combined tracks.

**Fig. 11:**
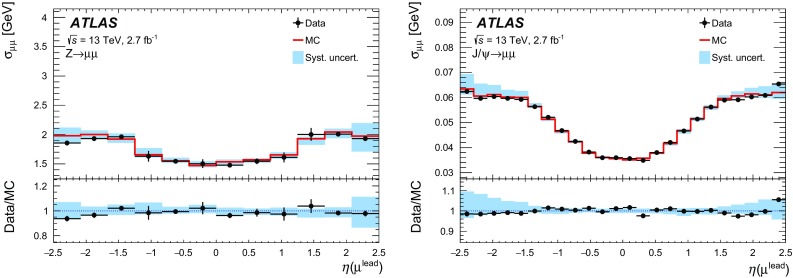
Dimuon invariant mass resolution for CB muons for ${Z\rightarrow \mu \mu }$ (*left*) and ${J/\psi \rightarrow \mu \mu }$ (*right*) events for data and corrected simulation as a function of the pseudorapidity of the highest-$p_{\text {T}}$ muon. The *upper panels* show the fitted resolution value for data and corrected simulation. The *lower panels* show the data/MC ratio. The *error bars* represent the statistical uncertainty; the *shaded bands* represent the systematic uncertainty in the correction and the systematic uncertainty in the extraction method added in quadrature

Figure 11 displays the dimuon mass resolution $\sigma (m_{\mu \mu })$ as a function of the leading-muon $\eta$ for the two resonances. The dimuon mass resolution is about 1.2 and $1.6~\%$ at small $\eta$ values for $J/\psi$ and *Z* bosons, respectively, and increases to 1.6 and 1.9 % in the endcaps. This corresponds to a relative muon $p_{\text {T}}$ resolution of 1.7 and $2.3~\%$ in the centre of the detector and 2.3 and 2.9 % in the endcaps for $J/\psi$ and *Z* boson decays, respectively. After applying the momentum corrections described above, the simulation reproduces the resolution measured in data, well within the systematic uncertainties. The systematic uncertainties are estimated following the same procedure described for the determination of the energy scale. Good agreement between the dimuon mass resolution measured in data and simulation is also observed for the ID and MS components of the combined tracks.

**Fig. 12:**
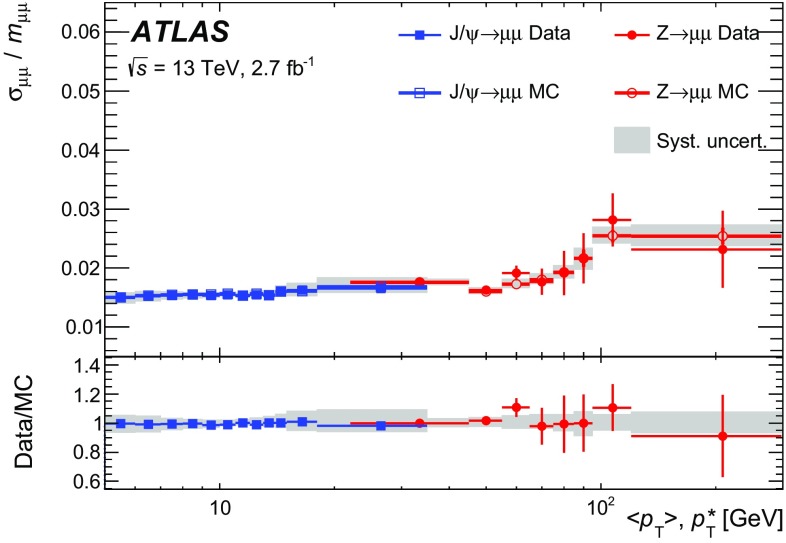
Dimuon invariant mass resolution divided by the dimuon invariant mass for CB muons measured from ${J/\psi \rightarrow \mu \mu }$ and ${Z\rightarrow \mu \mu }$ events as a function of the average transverse momentum variables $\langle {p}_\mathrm{T} \rangle$ and $p_{\text {T}} ^{*}$ defined in the text. Both muons are required to be in the same $|\eta |$ range. The *error bars* represent the statistical uncertainty while the bands show the systematic uncertainties

The relative dimuon mass resolution $\sigma _{\mu \mu }/m_{\mu \mu }$ depends approximately on the average momentum of the muons, as shown in Eq. (10). This allows a direct comparison of the momentum resolution function determined with $J/\psi$ and *Z* boson decays. This is shown in Fig. 12, where the relative dimuon mass resolution from ${J/\psi \rightarrow \mu \mu }$ and ${Z\rightarrow \mu \mu }$ events is compared to simulation. The ${J/\psi \rightarrow \mu \mu }$ and ${Z\rightarrow \mu \mu }$ resolutions are in good agreement. For the $J/\psi$, the average momentum is defined as $\langle p_{\text {T}} \rangle = \frac{1}{2}(p_{\mathrm {T},1}+p_{\mathrm {T},2})$ while for the *Z* boson it is defined as

12 $$\begin{aligned} p_{\text {T}} ^{*} = m_{Z} \sqrt{ \frac{\sin {\theta _1}\sin {\theta _2}}{2 (1-\cos \alpha _{12})}}, \end{aligned}$$

where $m_{Z}$ is the *Z* boson mass [30], $\theta _1$ and $\theta _2$ are the polar angles of the two muons, and $\alpha _{12}$ is the opening angle of the muon pair. This definition, based on angular variables only, removes the correlation between the measurement of the dimuon mass and the average $p_{\text {T}}$.

## Conclusions

The performance of the ATLAS muon reconstruction has been measured using 3.2 fb$^{-1}$ of data from *pp* collisions at $\sqrt{s} = 13$ TeV recorded during the 25 ns run at the LHC in 2015. A large calibration sample consisting of ${Z\rightarrow \mu \mu }$ decays and ${J/\psi \rightarrow \mu \mu }$ decays allows for a precise measurement of the reconstruction and isolation efficiency as well as of the momentum resolution and scale over a wide $p_{\text {T}}$ range.

The muon reconstruction efficiency is close to $99~\%$ over most of the pseudorapidity range of $|\eta |<2.5$ for $p_{\text {T}} >5$ GeV. The ${Z\rightarrow \mu \mu }$ sample enables a measurement of the efficiency with a precision at the 0.2 % level for $p_{\text {T}}$ $>$ 20 GeV. The ${J/\psi \rightarrow \mu \mu }$ sample provides a measurement of the reconstruction efficiency between 5 and 20 GeV with a precision better than 1 %.

The ${Z\rightarrow \mu \mu }$ sample is also used to measure the isolation efficiency for seven isolation working points in the momentum range 10–120 GeV. The isolation efficiency varies between 93 and $100~\%$ depending on the selection and on the momentum of the particle, and is well reproduced in the simulation.

The muon momentum scale and resolution have been studied in detail using ${J/\psi \rightarrow \mu \mu }$ and ${Z\rightarrow \mu \mu }$ decays. These studies are used to correct the simulation to improve the agreement with data and to minimise the systematic uncertainties in physics analyses. For ${Z\rightarrow \mu \mu }$ decays, the uncertainty in the momentum scale varies from a minimum of $0.05~\%$ for $|\eta | <1$ to a maximum of $0.3~\%$ for $|\eta |\sim 2.5$. The dimuon mass resolution is about $1.2~\%$ ($1.6~\%$) at small values of pseudorapidity for $J/\psi$ (*Z*) decays, and increases to 1.6 and 1.9 % in the endcaps for $J/\psi$ and *Z* decays, respectively. This corresponds to a relative muon $p_{\text {T}}$ resolution of 1.7 and $2.3~\%$ at small values of pseudorapidity and 2.3 and 2.9 % in the endcaps for $J/\psi$ and *Z* decays, respectively. After applying momentum corrections, the $p_{\text {T}}$ resolution in data and simulation agree to better than 5 % for most of the $\eta$ range.

## Footnotes

1 ATLAS uses a right-handed coordinate system with its origin at the nominal interaction point (IP) in the centre of the detector and the *z*-axis along the beam pipe. The *x*-axis points from the IP to the centre of the LHC ring, and the *y*-axis points upward. Cylindrical coordinates $(r,\phi )$ are used in the transverse plane, $\phi$ being the azimuthal angle around the beam pipe. The pseudorapidity and the transverse momentum are defined in terms of the polar angle $\theta$ as $\eta =-\ln \tan (\theta /2)$ and $p_{\text {T}} = p \sin \theta$, respectively. The $\eta$–$\phi$ distance between two particles is defined as $\Delta R=\sqrt{(\Delta \eta )^2 +(\Delta \phi )^2}$.
